# A nanoscale natural drug delivery system for targeted drug delivery against ovarian cancer: action mechanism, application enlightenment and future potential

**DOI:** 10.3389/fimmu.2024.1427573

**Published:** 2024-10-11

**Authors:** Yi Li, Qian Shen, Lu Feng, Chuanlong Zhang, Xiaochen Jiang, Fudong Liu, Bo Pang

**Affiliations:** ^1^ Guang’anmen Hospital, China Academy of Chinese Medical Sciences, Beijing, China; ^2^ First Clinical Medical College, Shandong University of Traditional Chinese Medicine, Jinan, China

**Keywords:** ovarian cancer, nanoparticles, nanoscale natural drug delivery system, targeted drug delivery, chemical compound

## Abstract

Ovarian cancer (OC) is one of the deadliest gynecological malignancies in the world and is the leading cause of cancer-related death in women. The complexity and difficult-to-treat nature of OC pose a huge challenge to the treatment of the disease, Therefore, it is critical to find green and sustainable drug treatment options. Natural drugs have wide sources, many targets, and high safety, and are currently recognized as ideal drugs for tumor treatment, has previously been found to have a good effect on controlling tumor progression and reducing the burden of metastasis. However, its clinical transformation is often hindered by structural stability, bioavailability, and bioactivity. Emerging technologies for the treatment of OC, such as photodynamic therapy, immunotherapy, targeted therapy, gene therapy, molecular therapy, and nanotherapy, are developing rapidly, particularly, nanotechnology can play a bridging role between different therapies, synergistically drive the complementary role of differentiated treatment schemes, and has a wide range of clinical application prospects. In this review, nanoscale natural drug delivery systems (NNDDS) for targeted drug delivery against OC were extensively explored. We reviewed the mechanism of action of natural drugs against OC, reviewed the morphological composition and delivery potential of drug nanocarriers based on the application of nanotechnology in the treatment of OC, and discussed the limitations of current NNDDS research. After elucidating these problems, it will provide a theoretical basis for future exploration of novel NNDDS for anti-OC therapy.

## 1 Introduction

Worldwide, OC is the leading cause of death from malignant tumors in the female reproductive tract. According to data released by the International Agency for Research on Cancer (1), there will be 313,959 new cases of OC worldwide in 2020, and this number will reach 348,000 cases by 2025. The incidence of OC is increasing at an alarming rate in many parts of the world, and the disease burden remains high, particularly in low Human Development Index countries lacking quality cancer treatment and care, with OC incidence and mortality well above the threshold assessed in the Global Cancer Report. Not only that, the annual “economic toxicity” of OC is also devastating, costing more than $5.8 billion annually in the United States alone (2). OC always develops in an unpredictable direction, and a series of symptoms such as lower abdominal and pelvic pain, irregular vaginal bleeding, and changes in bowel and urination habits occur insidiously with the progression of the tumor. OC is not only difficult to diagnose in the early stage, highly aggressive and prone to multi-drug resistance (MDR), but the survival rate of patients will drop sharply during stages III and IV. Metastatic OC is largely considered incurable, and some patients have a low response rate to single-target traditional therapies, hoping that traditional treatments (surgery, chemotherapy) can cure gynecological tumors has become as difficult as starting a broken “machine” (3). Although in recent years, researchers have hoped to use immunotherapy and polyADP-ribose polymerase inhibitors to reduce OC recurrence and chemotherapy resistance through “synthetic lethal” and “PARP capture”, the therapeutic effect of these therapies is still limited and the toxic side effects on the gastrointestinal tract and blood are large. No significant difference has been observed in the overall survival of patients (4, 5). Therefore, safer and more effective drug regimens are needed to improve patient outcomes.

Natural medicines include secondary metabolites, such as Triptolide (TP), Doxorubicin (DOX) and other compounds that are isolated and extracted from natural plants, animals, microorganisms and other organisms, having high efficiency and better biocompatibility compared with synthetic drugs. Studies have shown that 32% of small-molecule drugs approved globally in the last 40 years were derived from natural products and their derivatives (6). Natural drugs have complex structures and a wide variety, including polysaccharides, flavonoids, alkaloids, quinones, terpenoids, sterols, and so on. Interestingly, these natural substance components can transmit their functions on the organism itself to the human body, playing a role in signaling and improving immune levels. Therefore, all kinds of natural products have been widely used in nutritional supplements and treatment of inflammation, cancer, cardiovascular and other diseases (7, 8). However, just as a coin has two sides, most natural drugs have defects *in vivo* targeting, poor stability and low bioavailability, while new drug development also faces challenges such as lead optimization and identification of bioactive compounds, seriously hitting pharmaceutical companies’ enthusiasm for natural drug development (9). A reasonable scheme is to modify the chemical structure of natural drugs, which is to add artificial design at the level of biological macromolecules, improving the stability of natural drugs in the human body and the ability of targeted therapy. However, when it comes to bioavailability, researchers such as Tang (10) have found that artificial design at the biological macromolecule level may not be a good solution. Another hot idea, which has emerged in recent years, is to design targeted drug delivery strategies, such as relevant targeted delivery systems for OC therapy including peptide/folate/aptamer-drug conjugates, antibody-drug conjugates (ADCs), polymer-drug conjugates, ligand-functionalized nanomedicines, and dual-targeted nanomedicines, etc (11).

Among many drug delivery strategies, the nanoscale drug delivery system is a drug synergist that has emerged in recent years. As a multifunctional system, it can regulate the distribution of packaged drugs in time, space and dose (12). In the past few decades, nanotechnology has been absorbing technical solutions from different subdisciplines and has been widely used, especially in the fields of biology, medicine, and materials. Nanoscale materials which can be used as a delivery agent for encapsulating drugs or attaching therapeutic drugs, are materials with at least one dimension ranging from 1-100 nm in three-dimensional space, which have unique advantages in structural characteristics, chemical properties, mechanical dynamics, electrical performance, and biological properties (13, 14). Compared with traditional drug delivery methods, nano-scale materials can maximize the delivery and release of drugs at specific targets in the human body, reduce the adverse reactions brought by drugs, extend the blood circulation time of compounds, and have better bioutilization efficiency (15–17). Surprisingly, in recent years, researchers have modified nanomaterials onto biomolecules with the aid of aptamer conjugating technology, which has greatly improved the selective targeting ability of materials, providing new opportunities for tumor-targeted therapy. At present, natural drug delivery systems have been used in cancer treatment fields including OC, such as Liposome-DOX and albumin-bound paclitaxel (PTX) (18, 19). In addition, smart drug delivery platforms (20) have been developed in the field of nanocarriers, while clinical trials have demonstrated that nanoscale drug delivery systems are effective in improving the efficiency of cancer treatment (21). More importantly, nanoscale drug delivery systems offer new solutions to oncology drug treatment bottlenecks, including side effects, efficacy, availability, and targeting, compared to problems with traditional chemotherapy regimens.

In this study, we comprehensively review and summarize the current recommendations for NNDDS as a promising targeted therapy and drug delivery system for OC and provide a detailed summary evaluation of the morphological composition and delivery potential of drug nanocarriers. Meanwhile, we describe the mechanisms of action of natural drugs against OC, clarify the key principles of the natural drug delivery system, summarize the latest application status of NNDDS, pointing out recognized challenges and prospects in this promising area of research. The purpose of this study is to provide a basis for the research and development of NNDDS anti-OC targeted therapy.

## 2 Mechanism of action of natural drugs against OC

Currently, although the basic treatment options for OC are tumor reduction surgery and platinum-based chemotherapy, not all patients have the opportunity for surgery in clinical practice, and quite several patients are insensitive to chemotherapy or resistant to chemotherapy drugs in the later stage of treatment (22). In the early stages, even though the conventional treatment regimen is effective, the damage caused by the neurological and renal toxicity of chemotherapy drugs is difficult to estimate. At the same time, genetic mechanisms such as increased repair of DNA damage, disturbance of intracellular transduction pathways, and changes in cellular genes all act on the OC microenvironment and make the tumor resistant to drugs (23). Therefore, there is an urgent need to discover and develop new natural medicine preparations with alternative effects. In previous studies, we reviewed the independent predictive value of blood inflammatory complex markers for OC (24), and summarized and analyzed the specific mechanisms of natural polysaccharides against OC by intervening in inflammation and immune response, regulating tumor cell cycle, and affecting cell migration and invasion (25). However, in terms of the whole range of natural drugs, the mechanisms of anti-OC are more diverse, which is related to the complex and diverse structure of natural drugs (26). As expected, natural medicines remain an important source of compounds for the development of potential therapeutic agents for OC.

**Figure 1:**
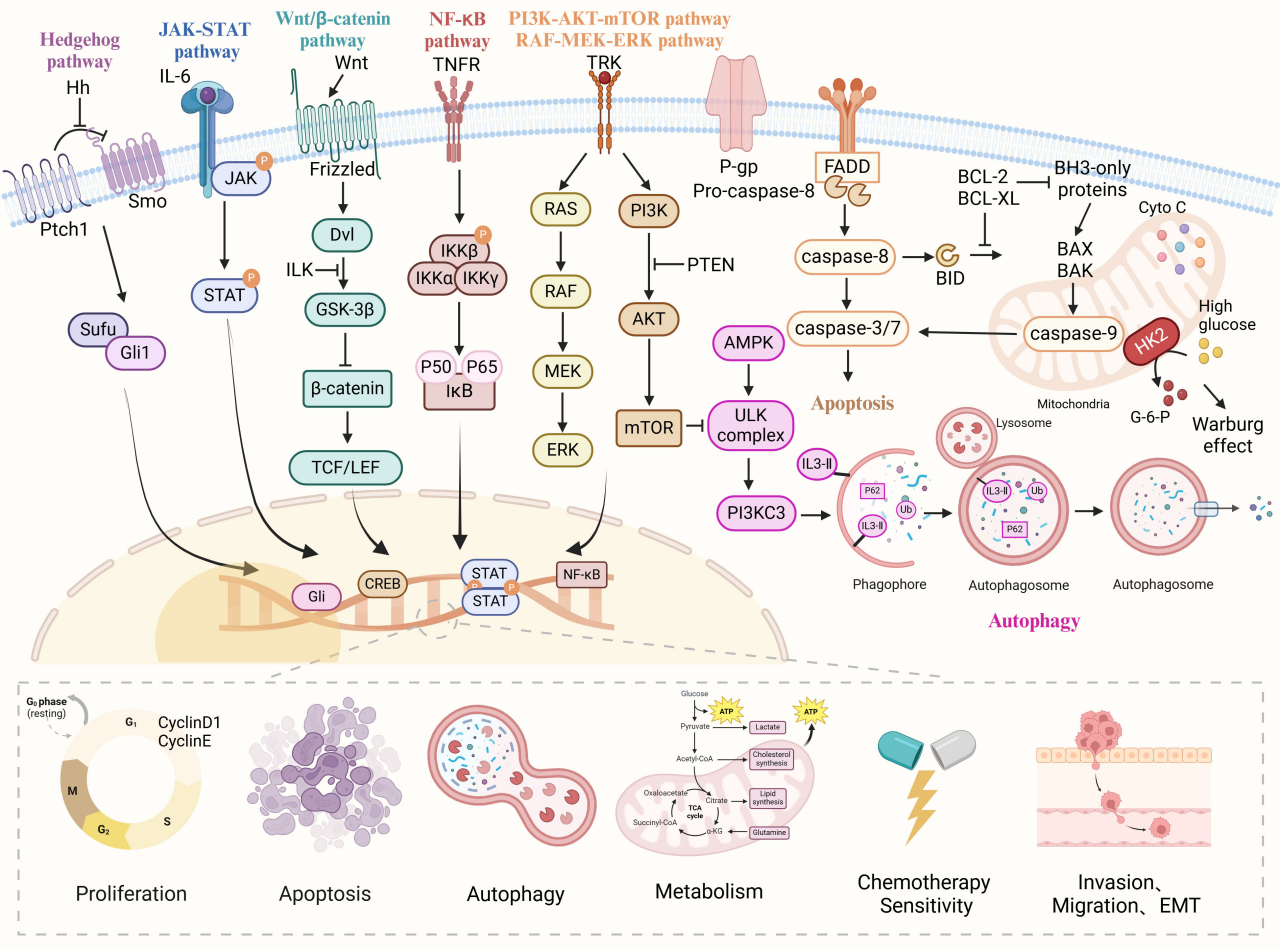
The various cellular signaling pathways of natural drugs in anti-ovarian cancer. Natural drugs through Hedgehog, JAK-STAT, Wnt/β-catenin, NF-κB, PI3K/AKT/mTOR, and Ras/Raf/MEK/ERK signaling pathways regulate cell proliferation, apoptosis, migration, invasion, EMT, autophagy, metabolism and chemotherapy sensitivity, thereby inhibiting the occurrence and development of ovarian cancer.

The biomolecular mechanism of anti-OC treatment with natural drugs can be divided into three parts, one is the direct treatment of OC cells, which means that natural drugs can inhibit the proliferation, invasion, and migration of tumor cells, block cell cycle and RNA expression, and induce apoptosis and autophagy; The second is the indirect anti-OC effect by improving the tumor microenvironment (TME), preventing neovascularization, regulating oxidative stress and DNA damage; The third is natural drugs as adjuvant chemotherapy drugs, can improve the sensitivity of chemotherapy drugs and reduce the side effects of chemotherapy drugs. The specific mechanism of natural products against ovarian cancer is shown in **Figure 1**.

### 2.1 The effect structure relationship of natural drugs against OC

An important characteristic of natural medicines compared to synthetic chemicals is that they contain a large variety of complex scaffolds, generally giving them higher molecular weight, more oxygen and carbon atoms, and fewer halogen and nitrogen atoms (27). Natural drugs against OC are mainly concentrated in flavonoids, polysaccharides, alkaloids, phenols, quinones, alcohols and other compounds, the rigid structure of which has a wide range of value in dealing with protein-protein interactions. This is often the key mechanism by which natural drugs exert their anti-tumor effects (28). Flavonoids are mostly low molecular weight compounds consisting of a tricyclic structure with various substituents, and their branching composition is often determined by the position of the intermediate epoxy group, the double bond between carbon atoms, or a partial hydroxyl group. These structures make it effective in anti-tumor proliferation, especially the hydroxylated structural pattern can increase the biological activity of flavonoids and promote the effect of inhibiting mast cell secretion (29, 30). In polysaccharides, the structure relationship of compounds is pretty clear, for example, most polysaccharides with anti-OC effects contain β-(1→3) -D-glucan main chain, and have similar alkaline glucan structure (31). It has been reported that β-(1→6)-glycoside and β-(1→3)-glycoside are the most active structures for anticancer activity, which may be related to the structural characteristics of the residual body (32). In alkaloids, the position of benzoyl, benzyl, and alkyl is related to their anti-tumor effect, and the structure that plays a key role or irreplaceable role in the compound is often called a “privileged scaffold”. Therefore, some active spatial structures are also regarded as the core content of the compound activity relationship (33–35). Phenols, quinones and alcohols are similar in structure, and there are few studies on their structural relationships. However, some studies have speculated that they all play similar roles in tumor prevention and regulation of carcinogen metabolism through hydroxymethyl-linked benzene rings (36, 37).

### 2.2 Natural drugs acting directly on OC cells

Berbamine, a bibenzyl isoquinoline alkaloid isolated from the plant *Berberis amurensis Rupr.* has previously been used for the treatment of leukemia in Asi (38). Zhang et al. (39) found that Berbamine could up-regulate the expression levels of caspase-3, caspase-9 and Bax by inhibiting the Wnt/β-catenin signaling pathway, inhibiting the proliferation activity of Human ovarian cancer SKOV-3 cells line(SKOV-3) and Human ovarian cancer ES-2 cells line(ES-2) cells. Baicalein is a natural medicine extracted from the root of Scutellaria baicalensis (SB). Yan et al. (40) demonstrated that Baicalein can inhibit the expression of matrix metalloproteinases (MMPs) in OC cells and significantly inhibit the invasion of OC cells. The biological mechanism may be related to the effect of Baicalein on p38 Mitogen‐activated protein kinase(MAPK)-dependent NF-κB signaling pathway. Scutellarein is another flavonoid substance derived from SB that has a different pharmacological effect due to the substitution of the hydroxyl group encoding position 7 in the molecular structure. Lang et al. (41) found that the proliferation rate of Human ovarian cancer A2780 cells line(A2780) and SKOV-3 cells treated with Scutellarein was significantly reduced, and the invasion and migration ability of OC cells were inhibited to the greatest extent when the concentration was 100 μM. In addition, many substances in SB, such as wogonin, Baicalin, Oroxylin A, etc. have been proven to be effective in the treatment of OC, suggesting that SB may be an important natural drug candidate for the treatment of OC.

The cell cycle, considered to be a key factor in tumor cell growth, is composed of G1, S, G2 and M cycles in mammals. Cyclin, cyclin-dependent kinase (CDK) and CDK inhibitors are the core substances that regulate the cell cycle. In OC, high expression of CDK 6 is often associated with a poor prognosis (42). Amentoflavone (AF) is a natural flavonoid derived from Selaginella tamariscina. Liu et al. (43) showed that AF can effectively inhibit the expression of S-phase kinase protein 2 (SKP2) in SKOV-3 cells and Human ovarian cancer OVCAR-3 cells line(OVCAR-3) cells through ROS/AMPK/mTOR signaling pathway, playing a role in blocking the cell cycle process. Astragalus polysaccharide (APS), a natural antitumor agent isolated from Astragalus membranaceus, has been shown to target the tumor suppressor F-box and WD-40 domain protein 7 through the regulation of miRNA, mediating miR-27a/FBXW 7 axis, which plays an anti-OC cell growth role *in vitro* (44). Deoxyschizandrin is the main active component of *Schisandra berries*, which has various biological activities such as hypoglycemic, antioxidant and anti-tumor. Lee et al. (45) found that Deoxyschizandrin can inhibit the expression of CD209 and CD163 in OC macrophages, promote the stagnation of the G_0_/G_1_ cell cycle of A2780 cells and play an anti-tumor role.

Apoptosis is an evolutionarily conserved form of programmed cell death in which cells actively die according to a specific genetic program to maintain the balance of tissues and organs. Apoptosis usually occurs in the process of organism development, tissue repair, immune response and tumor development (46). Zeylenone, a natural cyclohexanthin oxide derived from Uvaria grandiflora Roxb, was shown in a pilot study to reduce the expression of p-JAK and signal transduction and transcriptional activators of the Janus family of tyrosine kinases by increasing the mRNA levels of cytochrome C and apoptosis-inducing factors, promoting the apoptosis of SKOV-3 cells (47). Interestingly, the potential of Zeylenone to inhibit tumor cell proliferation and promote apoptosis has also been found in cervical cancer, of which the underlying mechanism is related to the inhibition of PI3K/AKT/mTOR and MAPK/ERK signaling pathways (48). Autophagy is a cell death pathway different from apoptosis. In the study of Che et al., the mechanism by which the natural metabolite grifolin induces autophagic death of OC cells has been extensively explored (49). The researchers treated A2780 cells and SKOV-3 cells with grifolin and found that it prevented tumor transformation at an early stage. Autophagy death of ovarian cancer cells was induced by up-regulating the expression of autophagy markers Beclin-1, LC3B, and Atg7, down-regulating the expression of P62, and inhibiting the expression of the Akt/mTOR/S6K pathway.

### 2.3 Natural drugs that act indirectly on OC cells

TME is a dynamic and complex acid-hypoxic system composed of malignant tumor cells, tumor-infiltrating immune cells, endothelial cells, extracellular matrix, vasculature, cytokines and chemokines. The mutual signaling between immune cells and OC can regulate the immune response and control disease progression (50). Natural drugs participate in multiple signaling pathways and molecular targets through metabolic crosstalk and indirectly act on tumor cells to exert anti-OC effects through metabolic regulation of TME cells. For example, resveratrol inhibits glycolysis levels and participates in regulating glycolysis and oxidation balance (51); Epigallocatechin gallate (EGCG) is one of the most bioactive catechins and has been found to have multi-target activity on endothelial cells. As revealed by the metabolic profile, EGCG interferes with the multi-pathway metabolism of tumor cells, and regulates oxidative stress and tumor angiogenesis, indirectly playing a role in anti-OC cell growth. Some studies integrating transcriptomics and metabolomics have also shown that EGCG has antioxidant stress effects on breast cancer and colon cancer (52, 53).

In recent years, the ability of OC cells to repair DNA damage has been regarded as an important sign of its refractory, therefore the intervention of natural drugs in the DNA damage repair pathway has become a hot target for OC treatment (54). Existing in a variety of natural plant alkaloid components, Berberine is a natural antioxidant and anti-inflammatory drug, that has been proven to be effective against lung cancer (55), stomach cancer (56), colorectal cancer (57) and other tumors. Hou et al. confirmed that berberine can significantly down-regulate homologous recombination repair of OC cells and induce oxidative DNA damage, and the experiment also shows that berberine can increase the sensitivity of A2780 cells and Human ovarian cancer HO8910 cells line(HO8910) cells to Niraparib, which is a natural drug with a wide range of targets for the treatment of OC (58).

### 2.4 Enhance sensitivity to chemotherapeutic drugs

Cisplatin and PTX or carboplatin and PTX adjuvant chemotherapy is the conventional treatment for OC patients after receiving cell reduction therapy. Unfortunately, more than half of the patients will relapse after treatment and develop platinum drug resistance, which poses no small challenge to the chemical treatment of OC. After drug resistance, tumor clinicians often choose the standard regimen of PEGylated liposome DOX in combination with weekly PTX and topotecan, however, its therapeutic effectiveness is often limited (59). In recent years, studies have found that natural drugs have sensitizing effects on chemotherapy drugs to a large extent, and their mechanism is related to regulating immune cell response, regulating the expression level of immune molecules, and reversing MDR (60). LycopeneIt, a natural red carotenoid found in tomatoes, has excellent antioxidant properties. Holzapfel et al. (61) found that Lycopene could significantly reduce cancer-related factors and metastasis load in mice with OC. The lycopene plus PTX regimen was as effective as the PTX plus platinum regimen in reducing OC burden compared with placebo. The mechanism is related to reducing the production of integrin α5β1 heterodimer, stimulating the proliferation of immune cells, and enhancing the activity of macrophages and T cells (62).

Grape seed procyanidin (GSP) is a natural polyphenol with good antioxidant properties. Zhao et al. extensively explored the cytotoxic effects of GSP on PTX and DOX against drug-resistant A2780/T cells (63). Studies have shown that GSP can reverse MDR phenotype by inhibiting P-glycoprotein (P-gp) function, not only enhancing the therapeutic effect of PTX and DOX for OC but also interacting with P-gp through MAPK/ERK pathway to block nuclear translocation. The mechanism of action of natural drug ingredients against OC in recent years is summarized in **Table 1**.

**Table 1:** Summary of experimental data on the mechanism of action of natural drug ingredients on OC.

		Phytochemicals	Phytochemical Source	Class	Molecular Formula	Molecular Weight (g/mol)	Ovarian cancer model	Mechanism	Effect	Reference
Direct Action		Anibamine	*Aniba panurensis*	Alkaloid	C_30_H_50_N^+^	424.7	OVCAR-3 cells	CCL5/CCR5 system	Inhibit growth	(64)
		Amentoflavone	*Selaginella tamariscina*	Flavonoid	C_30_H_18_O_10_	538.5	SKOV-3 and OVCAR-3 cells	Regulate ROS/AMPK/mTOR signaling pathway to inhibit the expression of SKP2	Induce cell cycle arrest	(43)
		Astragalus polysaccharide	*Radix Astragali*	Polysaccharide	C_10_H_7_ClN_2_O_2_S	254.7	OV-90, SKOV-3 and HEK 293T cells	Regulate miR-27a/FBXW7 axis	Inhibit proliferation and induce apoptosis	(44)
		Asiatic acid	*Centella asiatica*	Terpenoid	C_30_H_48_O_5_	488.7	SKOV-3 and OVCAR-3 cells	Inhibit PI3K/Akt/mTOR signaling pathway	Induce G_0_/G_1_ cell cycle arrest and apoptosis	(65)
		Berbamine	*Berberis amurensis*	Alkaloid	C_37_H_40_N_2_O_6_	608.7	SKOV-3 and ES-2 cells	Inhibit the Wnt/β-catenin signaling pathway and decrease caspase-3, caspase-9 and Bax level	Inhibit proliferative activity and promote apoptosis	(39)
		Baicalein	*Scutellaria baicalensis Georgi*	Flavonoid	C_15_H_10_O_5_	270.2	A2780/CP70, SKOV-3 and OVCAR-3 cells	Inhibit the expression of MMPs via p38 MAPK-dependent NF-κB signaling pathway; increased ROS production, DNA damage, and CHK2 upregulation and activation; Inhibit the expression of VEGF, HIF-1α, c-Myc, and NF-κB	Inhibit invasion and viability	(40, 66)
		Baicalin	*Scutellaria baicalensis Georgi*	Flavonoid	C_21_H_18_O_11_	446.4	OVCAR-3 and A2780/CP70 cells	Decrease the expression of VEGF; Attenuate YAP activity via inhibit RASSF6	Inhibit cell viability; Suppresses the cell stemness	(67, 68)
		Berberine	*Rhizoma coptidis*	Alkaloid	C_20_H_18_NO_4_^+^	336.4	OVCAR-3 and 3AO cells	Inhibit the expression of PCNA and Ki67 and enhanced the expression and activate the expression of Caspase-3, Caspase-8, RIPK3 and MLKL; Regulate miR-145/MMP16 axis; Down-regulate the EGFR-ErbB2/PI3K/Akt signaling pathway	Inhibit proliferation, migration and invasion; Promote apoptosis and cell necrosis; Induce G_0_/G_1_ cell cycle arrest	(69–71)
		Curcumin	*Amomum Tsao-Ko Crevostet*	Polyphenol	C_21_H_20_O_6_	368.4	PA-1, OVCAR-3, SKOV-3, HEY, OVCA42, OCC1 and MDAH2774 cells	Inhibit IL-6 and IL-8 secretion and STAT3 phosphorylation; Downregulate PIK3/AKT, increased caspase-3 and Bax, downregulated BCL2; Inhibit AKT phosphorylation and AKT protein, decreased the expression of BCL2 and surviving; Inhibit sarco/endoplasmic reticulum calcium ATPase and disrupted Ca^2+^ homeostasis; Upregulate miR-9 and modulate Akt/FOXO1 axis; Block stabilization of β1 integrin and consequently inhibit FAK and EGFR activation; Regulate circ-PLEKHM3/miR-320a/SMG1 axis; Activate the NRF2/ETBR/ET-1 Axis;	Inhibit cell motility, EMT, proliferation, cell invasion potential; Induce G2/M cell cycle arrest and apoptosis; cytotoxic effects	(72–78)
		Cucurbitacin-A	*Cucumis sativus*	Terpenoid	C_32_H_46_O_9_	574.7	SKVO-3 cells	Inhibit mTOR/PI3K/Akt signaling pathway	Induce G2/M cell cycle arrest, DNA damage and prompted ROS-mediated alterations in MMP	(79)
		Carnosol	*Radix Salviae*	Terpenoid	C_20_H_26_O_4_	330.4	SKOV-3, OVCAR-3 cells	Inhibit the EGF-induced EMT	Reduce cell viability and inhibit cell proliferation and EMT	(80)
		Dihydroartemisinin	*Artemisia annua*	Terpenoid	C_15_H_24_O_5_	284.4	SKOV3, SKOV3-IP, HO8910 and HO8910-PM cells	Inhibit the hedgehog signaling pathway	Induce apoptosis and inhibit proliferation, migration, and invasion	(81)
		Epigallocatechin Gallate	*Ginkgo Semen*	Flavonoid	C_22_H_18_O_11_	458.4	A2780, SKOV-3, OVCAR-3 and PA-1 cells	Regulate the expression of Bax, p21, Retinoblastoma, cyclin D1, CDK4, PCNA and Bcl-X; Down-regulate expression of AQP5 and NF-κB; Induce the activation of FOXO3A and suppressed the expression of c-Myc; Regulate PTEN/AKT/mTOR signaling pathway	Induce apoptosis and G1 or G1/S cell cycle arrest; Inhibit the proliferation	(82–85)
		Emodin	*Rheum palmatum*	Quinones	C_15_H_10_O_5_	270.2	A-2780 and SKOV-3 cells	ILK/GSK-3β/Slug Signaling Pathway	Inhibit EMT	(86)
		Formononetin	*Dalbergia, Glycyrrhiza pallidiflora*	Flavonoid	C_16_H_12_O_4_	268.3	ES2 and OV90 cells	Inhibit PI3K/AKT and ERK1/2	Induce apoptosis and G_0_/G_1_ cell cycle arrest	(87)
		Icaritin	*Epimedium*	Flavonoid	C_21_H_20_O_6_	368.4	A2780 and A2780cp cells	Inhibit the Akt/mTOR signaling pathway	Inhibit migration and invasion	(88)
		Ginsenosides	*Panax ginseng*	Terpenoid	C_30_H_52_O_2_	444.7	SKOV-3, 3AO and A2780 cells	Reduces the expression of HIF-1α by activating the ubiquitin-proteasome pathway; Attenuate the expression of NF-κB and HIF-1α; Inhibit miR-25/EP300/E-cadherin pathway; Inhibit the expression of lncRNA H19; Abrogate KIF20A overexpression-induced CDC25A upregulation.	Inhibit EMT, migration, proliferation and invasion.	(89–93)
		Isoliquiritigenin	*Glycyrrhiza glabra*	Flavonoid	C_15_H_12_O_4_	256.3	OVCAR5 and ES-2 cells	Increase cleaved caspase-3, cleaved PARP, Bax/Bcl-2, LC3B-II and Beclin-1 levels	Inhibit growth, Induce G2/M cell cycle arrest, apoptosis and autophagy	(94)
		Oroxylin A	*Scutellaria baicalensis Georgi*	Flavonoid	C_16_H_12_O_5_	284.3	SKOV-3 cells	Increase PPARγ expression and alter the expression of PGRMC1/2	Inhibit migration and cell viability	(95)
		Deoxyschizandrin	*Schisandra berries*	Lignan	C_24_H_32_O_6_	416.5	A2780, SKOV-3 and OVCAR-3 cells	Inhibit Cyclin E and the M2 phenotype markers CD163 and CD209 expression	Induce G_0_/G_1_ cell cycle arrest and reduce the protumoural phenotype of tumor-associated macrophages	(45)
		Harmine	*Tribulifructus*	Alkaloid	C_13_H_12_N_2_O	212.3	SKOV-3 cells	Inhibit the ERK/CREB pathway	Inhibit proliferation and migration	(96)
		Grifolin	*Albatrellus confluens*	Terpenoid	C_22_H_32_O_2_	328.5	A2780 and SKOV-3 cells	Down-regulate Akt/mTOR/S6K signaling pathway	Inhibit proliferation; Induce autophagic cell death	(49)
		Kudsuphilactone B	*Schisandra chinensis*	Terpenoid	-	-	A2780 cells	Stimulate the activation of caspase-3, -8, and -9	Induce caspase-dependent apoptosis	(97)
		Tetramethylpyrazine	*Ligusticum wallichii Franch*	Pyrazine	C_8_H_12_N_2_	136.2	SKOV-3 cells	Decrease the expression of IL-8 via the ERK1/2, p38 and AP-1 signaling pathway	Inhibit migration	(98)
		Quercetin	*Ginkgo Semen*	Flavonoid	C_15_H_10_O_7_	302.2	PA-1, A2780 and SKOV-3 cells	Activate the extrinsic death receptor-mediated and intrinsic mitochondrial apoptotic pathway; Induce mitochondrial-mediated apoptotic pathway; Promote TRAIL-mediated apoptosis via upregulating the transcription of DR5.	Inhibit proliferation; Cause cell cycle arrest; Induce apoptosis	(99–102)
		Resveratrol	*Mori Cortex*	Polyphenol	C_14_H_12_O_3_	228.2	OVCAR-3, A2780 and SKOV-3 cells	Down-regulate Akt/GSK and ERK signaling pathway and decrease the protein cyclinD1 level; Decrease cellular α5β1 integrin level by increasing the secretion of hyaluronic acid; Decrease HIF-1α and VEGF via p42/p44 MAPK pathway; Activate the p38 MAPK and suppress AKT	Induce cell cycle arrest apoptosis; Inhibit proliferation, adherence, migration	(103–105)
		Sulforaphane	*Brassica oleracea*	Isothiocyanate	C_6_H_11_NOS_2_	177.3	OVCAR-3, OVCAR-4, OVCAR-5 and SKOV-3 cells	Promote ROS damage	Inhibit growth	(106)
		Sideroxylin	*Callistemon lanceolatus*	Polyphenol	C_18_H_16_O_5_	312.3	ES2 and OV90 cells	Induct mitochondrial dysfunction and the activation of PI3K and MAPK signaling pathway	Inhibit proliferation	(107)
		Scutellarein	*Scutellariae Radix*	Flavonoid	C_15_H_10_O_6_	286.2	A2780 and SKOV-3 cells	Regulate EZH2/FOXO1 signaling pathway	Inhibit proliferation, migration, and invasion	(41)
		Sanguiin H-6	*Rubus idaeus*	Polyphenol	C_82_H_54_O_52_	1871.3	A2780 cells	Regulate the MAPK p38 and a caspase-8-dependent BID cleavage pathway	Inhibit proliferation and induce apoptotic cell death	(108)
		Wogonin	*Forsythiae Fructus*	Flavonoid	C_16_H_12_O_5_	284.3	CaOV3 and A2780 cells	Downregulate protein levels of ER-α, VEGF, Bcl-2, Akt, increased expressions of Bax, p53 and caspase-3; Decrease catalase activity and raise intracellular hydrogen peroxide; Inhibit the activity of Indoleamine 3,5-dioxygenase-1 and down-regulate the expression of VEGF	Inhibit proliferation, decrease the percentage of G_0_/G_1_ and reduce invasiveness; Improve tumor necrosis factor-induced apoptosis; Improve the immunosuppressive environment, reduce angiogenesis and migration	(109–112)
		Zeylenone	*Uvaria grandiflora*	Terpenoid	C_21_H_18_O_7_	382.4	SKOV-3 cells	Decrease the expression of p-JAK and p-STAT, increase Caspase-3, Fas, FasL, Bax, Cyto c and AIF	Inhibit proliferation and promote apoptosis	(47)
		Proanthocyanidins	*Cranberry; Pinus massoniana bark*	Polyphenol	-	-	SKOV-3, A2780 and OV2008 cells	Block vascular endothelial growth factor-stimulated receptor phosphorylation; Down-regulate the activation of Bcl-2, Caspase 3/9, MMP-9, NF-κB, ERK1/2 and p38 MAPK	Anti-angiogenesis and cytotoxicity; Induct apoptosis and Inhibit migration	(113, 114)
Indirect Action	Autophagy	Resveratrol	*Mori Cortex*	Polyphenol	C_14_H_12_O_3_	228.2	A2780, CaOV3, ES-2, TOV112D, A1947, SKOV-3, NUTU-19 and A2780 cells	Release of cytochrome c and activation of caspase 9; Inhibit STAT3, increase LC3 and Beclin-1 levels; Hedgehog pathway; Induce ROS and Atg5 expression.	Induce autophagy	(115–119)
		Withaferin-A	*Withania somnifera*	Lactone	C_28_H_38_O_6_	470.6	A2780, A2780/CP70 and OVCAR-3 cells	Inhibit the expression of autophagy marker LC3B, accumulation of reactive ROS	Induce autophagy and DNA damage	(120)
		Curcumin	*Amomum Tsao-Ko Crevostet*	Polyphenol	C_21_H_20_O_6_	368.4	SKOV-3 and A2780 cells	Inhibit AKT/mTOR/p70S6K pathway	Induce autophagy	(121)
		Berberine	*Rhizoma coptidis*	Alkaloid	C_20_H_18_NO_4_^+^	336.4	SKOV-3, OVCAR-3, A2780, HEY, HO8910 and HO8910PM cells	Increase ROS expression, and downregulate RAD51 expression and homologous recombination repair	Induce oxidative DNA damage and impair homologous recombination repair	(58)
		Ginsenosides	*Panax ginseng*	Terpenoid	C_30_H_52_O_2_	444.7	SK-O-V3 cells	Upregulate LC3, Atg5 and Atg7	Induce autophagy	(122)
	metabolism	Ginsenosides	*Panax ginseng*	Terpenoid	C_30_H_52_O_2_	444.7	SKOV-3 cells	Regulate STAT3/HK2 pathway; Regulate H19/miR-324-5p/PKM2 pathway; Up-regulate miR-603 and down-regulate HK2	Inhibit the Warburg effect;	(123–125)
		Resveratrol	*Mori Cortex*	Polyphenol	C_14_H_12_O_3_	228.2	SKOV-3, OVCAR-5 and ID8 cells	Reduce glucose uptake by tumors; Interrupt protein glycosylation; Reduce lactate production; Regulate glycolysis and oxidation balance	Inhibit glucose metabolism	(51, 126–128)
		Berberine	*Rhizoma coptidis*	Alkaloid	C_20_H_18_NO_4_^+^	336.4	SKOV3 and 3AO cells	Regulate TET3/miR-145/HK2 pathway	Antagonize the Warburg effect	(129)
Enhanced Chemotherapy Sensitivity		Curcumin	*Amomum Tsao-Ko Crevostet*	Polyphenol	C_21_H_20_O_6_	368.4	A2780, A2780cp, A2780-cis, SKOV3, SKOV3/Txr, MDAH2774 and 2774/Txr cells	Restore MEG3 levels via demethylation and decrease EVs mediated transfer of miR-214; Inhibit PI3K/AKT/mTOR pathway; Upregulate SNIP1 and inhibit NF-κB activity; Regulate the miR-9-5p/BRCA1 axis	Induce chemo/radio-sensitization	(130–134)
		Lycopene	*Solanum lycopersicum*	Terpenoid	C_40_H_56_	536.9	OV-MZ-6 cells	Reduce the production of integrin α5β1 heterodimer, reduce activation of MAPK, stimulate the proliferation of immune cells, enhance the activity of macrophages and T cells	Enhance anti-tumorigenic effects of paclitaxel and carboplatin	(61)
		Resveratrol	*Mori Cortex*	Polyphenol	C_14_H_12_O_3_	228.2	A2780 cells	Inhibit PI3K/AKT/mTOR pathway; Downregulate NF-κB; Modulate the EGFR or VEGFR family of receptor tyrosine kinases.	Reduce resistance to platinum	(132, 135)
		Quercetin	*Ginkgo Semen*	Flavonoid	C_15_H_10_O_7_	302.2	SKOV-3/CDDP cells	Induce ROS production and the mitochondrial apoptotic pathway	Improve cisplatin resistance	(136)
		Baicalein	*Scutellaria baicalensis Georgi*	Flavonoid	C_15_H_10_O_5_	270.2	A2780 and A2780/CDDP	Regulate CirSLC7A6/miR-2682-5p/SLC7A6 axis.	Improve the chemoresistance	(137)
		Berberine	*Rhizoma coptidis*	Alkaloid	C_20_H_18_NO_4_^+^	336.4	OV2008, A2780, A2780/DDP, SKOV-3 and OVCAR-3 cells	Interfere the expression of dihydrofolate reductase and thymidylate synthase; Regulate miR-21/PDCD4 axis; Regulate miR-93/PTEN/AKT axis; Suppress the arachidonic acid metabolic pathway and phosphorylation of FAK; Inhibit chemotherapy-activated GLI1/BMI1 signaling pathway.	Increase sensitivity to cisplatin; inhibit the chemotherapy‐induced repopulation; inhibit chemotherapy-exacerbated cancer stem cell-like characteristics and metastasis	(138–141)
		Oroxylin A	*Scutellaria baicalensis Georgi*	Flavonoid	C_16_H_12_O_5_	284.3	SKOV-3 cells	Decrease P-gp-mediated drug efflux	Improve the sensitivity of chemotherapy drugs	(142)
		Epigallocatechin Gallate	*Ginkgo Semen*	Flavonoid	C_22_H_18_O_11_	458.4	OVCAR-3, SKOV-3, A2780 and A2780/CP20 cells	Enhance cisplatin-induced apoptosis, G2/M phase arrest and upregulate p21 expression; Inhibit the rapid degradation of Ctr1 induced by cisplatin; Down-regulate hTERT and Bcl-2 and promote DNA damage	Enhance the efficacy of cisplatin; Induce paclitaxel-resistant cell apoptosis	(143–145)
		Wogonin	*Scutellaria baicalensis Georgi*	Flavonoid	C_16_H_12_O_5_	284.3	SKOV3, SKOV3/DDP, OV2008, C13, A2780 and PTX10 cells	Up-regulate p53 protein and down-regulate Akt protein; Inhibit the PI3K/Akt pathway	Develop resistance to carboplatin and paclitaxel; Increase cisplatin sensitivity;	(146, 147)
		Proanthocyanidins	*Myrica rubra Sieb. et Zucc; Vitis vinifera*	Polyphenol	C_30_H_26_O_13_	594.5	A2780/CP70 A2780/T, A-2780 and SKOV-3 cells	Regulate Wnt/β-catenin, AKT/mTOR/p70S6K/4E-BP-1 signaling pathway; Down-regulate NF-κB and MAPK/ERK to mediate YB-1 activity	Inhibit angiogenesis and induce G1 cell cycle arrest; Reverse p-glycoprotein associated multi-drug resistance	(63, 148–150)
		Voacamine	*Peschiera fuchsiaefolia*	Alkaloid	C_43_H_52_N_4_O_5_	704.9	A2780 DX	Interfere with the P-gp function and inhibit the nuclear translocation of NF-kB	Evaluate the chemosensitizing effect	(151)
		Toosendanin	*Melia toosendan*	Terpenoid	C_30_H_38_O_11_	574.6	SKOV-3 and SKOV-3/DDP cells	Modulate the miR-195/ERK/β-catenin pathway	Reduce cisplatin resistance	(152)
		Glaucocalyxin B	*Rabdosia japonica*	Terpenoid	C_22_H_30_O_5_	374.5	A2780 and A2780/DDP cells	Increase ROS level, the phosphorylation of JNK and DNA damage	Attenuate growth and cisplatin resistance	(153)
		Ginsenosides	*Panax ginseng*	Terpenoid	C_30_H_52_O_2_	444.7	OVCAR-8, SKOV-3, HEYA8NCI/ADR-RES and DXR cells	Inactivate P-gp and Src inhibition; Inhibit Wnt/β-catenin pathway and EMT.	Reverse multidrug resistance	(154, 155)

## 3 Application advantages of NNDDS in the treatment of OC

### 3.1 Improve the ability to target drug delivery to OC

The development of NNDDS enables targeted delivery of hydrophobic components of natural drugs driven by specific ligand functions, preferentially acting on OC cells and releasing the drug into tumor tissue. Nanoscale delivery systems enable better biocompatible distribution and pharmacokinetics of natural drugs compared to conventional delivery modes and amplify the therapeutic potential of natural drugs compared to non-functional delivery modes (156). To verify the effect of the nanoscale drug delivery system, Vandghanooni et al. (157) prepared nanoparticles of AS1411 anti-riboprotein-aptamer modified PEGylated poly (lactic-co-glycolic acid) and loaded them with a chemotherapeutic agent. Compared with traditional drug delivery methods, functionalized nanoparticles can strongly inhibit endogenous miR-21 expression through endocytosis. Therefore, targeted delivery of therapeutic drugs to Mir-21-inhibited OC cells using nanomedicine delivery systems can improve the mortality of tumor cells. Fang et al. (158) modified the amphiphilic block copolymer Pluronic F68 with conjugated linoleic acid, synthesized the F68-Linoleic acid (F68-LA) conjugate, and formed a drug delivery system by loading the natural drug Gambogic acid (GA), improving the therapeutic effect of OC. The study found that compared with the control group, the particle size and potential of GA-loaded F68-LA nano-spheres could remain unchanged for up to 6 days, and the cytotoxicity and pro-apoptotic effect of GA-loaded F68-LA nano-spheres was significantly enhanced on A2780 cells, which not only had high stability but also had the ability of targeted therapy on tumors. All these results suggest that nanoscale drug delivery systems are a promising prospect for therapeutic targeted intervention in OC.

### 3.2 Prolong the blood circulation time of the drug

Nanostructured carriers deliver drugs mainly through active delivery and passive delivery, in which drugs integrate into the inner cavity of the nanostructure with the help of hydrophobic effect, and realize the transfer of drugs from high concentration to low concentration at specific targets, and then release a specific amount of drugs. Meanwhile, nanomedicine materials designed in conjugates disengage from the carrier very quickly, making drug delivery easier (159). NNDDS changes the original pharmacokinetic characteristics of natural drugs to a certain extent and regulates the blood circulation of drugs by regulating the mechanism and rate of drug release reaction, improving the tumor treatment effect of nano combination (160). Yao et al. (161) have developed a novel targeted PTX-supported nanocore PTX-PEG-PLA-FA-NP for OC treatment, which connects Folic acid (FA) molecules to PEG-PLA to form block copolymers through covalent bonds between the hydroxyl group of FA molecules and the ends of polyethylene glycol and polylactic acid (PEG-PLA). SKOV-3 cells, HO8910 cells, and A2780 cells were used in the PTX-PEG-PLA-FA-NP to measure drug release and uptake *in vitro*, and pharmacokinetics and drug distribution *in vivo* were studied by high-performance liquid chromatography (HPLC). Ptx-peg-pla-fa-np has a stronger uptake of SKOV-3 cells *in vitro* than free PTX. Relevant studies on drug distribution *in vivo* confirmed that compared with the normal administration group, PTX loaded with nanocarriers contained 3 times more PTX drug concentration in tumor tissues, and had longer blood circulation time, playing a perfect drug release and anti-OC treatment at the same drug dose.

## 4 Types and characteristics of NNDDS in anti-OC therapy

As mentioned in the previous introduction, NNDDS have shown a powerful function in the delivery of natural drugs to exert anti-tumor effects, not only improving the ability of targeted drug delivery to OC, but also controlling drug release, extending the blood circulation time of the drug, and maximizing the anti-tumor therapeutic effect. At present, the anti-tumor drug delivery of nano drug delivery system is mainly through active targeting and passive targeting. Active targeting uses the principle of ligand-receptor recognition to deliver natural or synthetic drugs to cells that promote drug capture and have specific receptor functions (162). Disorder and dilation are the normal conditions of new blood vessels, as the dense blood vessels of the tumor, the space of the blood vessel wall is relatively wide, and the phenomenon of lymphatic reflux is obstructed. This pathophysiological feature of tumors has selectivity and retention of macromolecular substances, in such cases, passive targeting came into being. Passive targeting refers to relying on the unique properties of nanoscale carriers to improve the permeability of drugs to tumor tissues so that drug concentration can accumulate. At the same time, the change of pathological microenvironment can reduce the kidney clearance rate of drugs, so that therapeutic drugs can enter and stay in tumor tissues (163). Physical encapsulation and front-end carrier connection are two major strategies for drug delivery systems. Physical encapsulated drug delivery systems include liposomes, polymer micelles, polymer-drug conjugates, nanosuspensions, etc., which are mostly associated with passive targeted drug delivery modes. The front-end carrier-linked drug delivery system involves ligands with high adaptation to tumor cell differentiation, of which the acceptors include FA receptors, EGFR receptors, HER2 receptors, etc., associated with active targeted drug delivery modes.

### 4.1 Lipid-based drug delivery systems

Lipid-based nanodrug delivery systems are mainly composed of liposomes and solid lipid nanoparticles (SLNs), currently widely used delivery carriers of lipid-based DDS. The combination with drugs is also the most common type of nanomedicine formulation currently approved (164). Liposome nanoparticles have long been regarded as carriers for the transport and management of various drugs into cells, and like other nanotechnology platforms, they can selectively improve the distribution and release efficiency of natural drugs *in vivo*. Liposome nanoparticles come from a wide range of sources and are the most used type in current tumor clinical trials, accounting for about 33% (165). The discovery of EXO, which are complex versions of liposomes, has led to advances in models of personalized medicine, especially in the fields of cancer immunotherapy and complementary and alternative medicine. It has received considerable attention, providing a broader perspective for the study of the biological activity, substantive capacity and targeted drug delivery of liposome nanoparticles in diseases (166). As lipids have the characteristics of hydrophilic head and hydrophobic tail, they can be used to deliver hydrophilic and hydrophobic drugs, and have a wide application prospect.

#### 4.1.1 Liposomes drug delivery system

##### 4.1.1.1 Natural drug-loaded liposomes: extracellular vesicle

EV, composed of proteins such as four-transmembrane protein family, cellular endogenous proteins, skeleton proteins, non-coding RNA, mRNA and other substances, and bioactive lipids, is a nanoscale vesicular body with a diameter of about 20-200nm that is released by cells into the extracellular matrix. Now seen as a key mediator that plays an important role in intercellular communication, there is growing evidence that EVs are an important tool for targeted therapy of disease (167). In terms of drug delivery, EV has the advantages of strong biobarrier permeability, high biocompatibility, low toxicity and low immunogenicity, and is a natural lipid nanoparticle. EV plays a role in drug delivery mainly through receptor and ligand interaction, membrane fusion, cell phagocytosis and other forms, to transfer bioactive therapeutic drugs from the supplier to a specific target cell (168). According to the diameter and origin, EV can be divided into exosomes (EXO), microvesicles (MVS), apoptotic bodies, phagosomes, etc. EXO, a double-layer membrane-structure vesicle formed by the fusion of polyvesicles and plasma membrane, is formed from the plasma membrane in the way of budding, and apoptotic bodies are released after apoptosis.

###### 4.1.1.1.1 EXO

EXO is relatively stable and widely sourced in circulation and can be selectively captured by tumor cells. It only plays the role of transportation like presenting “express parcels” without changing the function of the original drugs, as a promising nanoscale drug delivery carrier. In OC, EXO is often considered a biomarker of ovarian metastasis. According to the National Cancer Institute, 213 proteins are shared across 60 tumor cell lines from nine different cancers (93). In general, EXO derived from dendritic cells, macrophages, mesenchymal stem cells, B cells, and T cells can often be used as drug-carrying EXO, further promoting the application of natural drugs in anti-tumor therapy (169). The parental cell characteristics of EXO are reflected in surface markers, contents and cell activation pathways. It has significant advantages in the targeted recognition of tumor cells while avoiding systemic inflammation and immune system rejection (170).

Berries are rich in bioactive substances, especially anthocyanins with antioxidant, anti-inflammatory, and anti-tumor properties, which have been widely recognized for their importance to health. In the study of Munagala et al. (171), berry anthocyanins have anti-tumor cell proliferation effects on various cancer types such as lung, prostate, colon, breast, pancreas, and ovary. The antitumor function of anthocyanins *in vitro* has been fully explored, therefore anthocyanins have the potential to be developed as a therapeutic agent for OC. The daily dose required to supplement anthocyanins with berry intake is considerable, which is also impractical to scale up conversion in a population of patients with weakened OC (172). In addition, as there is ample evidence that oral anthocyanins have low bioavailability and stability, it is often futile to rely on free anthocyanins for the treatment of OC patients (173). In order to overcome these limitations, Farrukh et al. developed anthocyanins delivered via milk-derived EXO, including improving the passive diffusion absorption and availability of anthocyanins and using natural medicines to reduce platinum resistance in some OC patients (174). Studies have shown that even a suboptimal dose of anthocyanin EXO can significantly inhibit OC growth in nude mice, with better stability, higher cell uptake capacity and longer blood circulation time than free anthocyanin. In addition, this study also found that anthocyanin EXO preparation can reduce the corresponding chemotherapy dose threshold when OC cells develop cisplatin resistance, having the potential value of sensitizing drug-resistant OC cells.

TP is the main pharmacodynamic component of natural Chinese herbal medicine. Previous studies have found that TP can induce lethal autophagy and sensitizing chemotherapy drugs for cisplatin-resistant OC, and reduce the growth and metastasis of solid tumors. Therefore, to improve the poor solubility of TP, cytotoxic compounds, Liu et al. (175) developed a new bio-derived nanomaterial, Triptolide-loaded exosomes delivery system (TP-EXOS), a drug delivery system using EXO as a delivery carrier. This study evaluated the anti-OC effect of TP-EXOs *in vivo* and *in vitro* by nanoparticle tracking analysis, high-performance liquid chromatography, BrdU/DNA two-parameter flow cytometry, and other technologies. The results showed that TP-EXOS could block OC cells in the S phase, and the effect of inhibiting the proliferation of SKOV-3 cells was two times higher than that of carrier-free TP therapy. At the same time, TP-EXOs have a higher drug encapsulation rate, which can enrich natural drugs to the tumor site of tumor-bearing mice, and play an anti-OC role. Unfortunately, although the exosome delivery vector has endowed TP with the ability of targeted therapy, TP-EXOs also weaken the cytotoxicity and apoptosis-inducing effect of TP on SKOV-3 cells to a certain extent and does not completely reduce the liver toxicity of TP, which still needs to be further optimized around these problems to achieve the best therapeutic effect in the future.

In the study of Melzer et al., researchers isolated EXO containing PTX from mesenchymal stromal cells (MSC) treated with sublethal concentrations of PTX for 24 hours and applied it to lung, ovarian, and breast cancer cells (176). It was found that EXO containing PTX could play a specific role in drug delivery and accumulation in tumor tissues. Compared to the control group, the drug delivery system showed a strong tumor cell killing effect even with a 7.6-fold reduction in dose, demonstrating the usefulness of EXO derived from MSC to serve as a drug delivery vehicle to deliver chemotherapy compounds to multiple cancer cells.

###### 4.1.1.1.2 MVS

MVS, a class of membranous vesicles ranging in diameter from 100 to 1000 nm, are released by the cell via budding during activation, injury, or apoptosis. Unlike EXO, MVS originates directly from the plasma membrane and is released into the extracellular space through a series of division processes (177, 178). As an important tool for intracellular communication *in vivo*, the unique bilayer phospholipid structure of MVS can effectively protect chemotherapy drugs in MVS from being metabolized before reaching the tumor site. In addition, MVS are excellent vectors for drug delivery, with good *in vivo* circulation stability, tumor targeting, physiological barrier permeability, and almost no immunogenicity and toxicity. Currently, in NNDDS for OC, there are few types of MVS as carriers, which may be related to the fact that MVS as drug carriers is still a new research field. At present, most of the existing studies on MVS as drug carriers focus on pancreatic cancer (179) and colon cancer (180), and the experimental results of different tumor cells have shown that MVS has good stability and immune tolerance. The existing experimental results have paved the way for MVS as an NNDDS for OC, which is worth looking forward to further research.

##### 4.1.1.2 Synthetic drug loaded liposomes

Chrysin, a natural flavonoid derived from *Passiflora caerulea L., Oroxylum indicum (L.) Kurz and other plant sources*, has previously been shown to have anti-tumor, anti-inflammatory and antioxidant effects. Chrysin and its derivatives have good anticancer activity against a variety of cancers. For example, the killing activity of its long-chain ester derivatives on liver cancer cell lines is 5.4 times that of Chrysin, and its porphyrin derivatives can be used for non-invasive photodynamic therapy of cervical cancer and gastric cancer (181). Chrysin is not an excellent drug in terms of its low bioavailability in the body, as low bioavailability is often indicative of low performance. In order to ameliorate this defect, a common approach is to produce flavonoid derivatives by biotransformation, such as microbial and enzymatic reactions, which does not substantially change their pharmacological action, but significantly improves their bioavailability due to structural changes. Although this recognized technique improves the performance of Chrysin, it is not the best solution to improve the bioavailability of Chrysin due to the limitations of complex technology and high cost (182). In order to solve the defects of poor absorption and rapid metabolism of Chrysin applications, Tarahomi et al. (183) designed and synthesized nanoliposomes *Nano-niosomes containing chrysin* (Chr-NiO) containing poplar. The liposomal drug delivery system was tested to improve the conversion performance in Chrysin and the toxic effect of Chr-NiO on SKOV-3 cells. The experimental results showed that, compared with conventional drugs, CR-NiO significantly inhibited the migration of SKOV-3 cells *in vitro* scratch test. The expression of the apoptosis gene in SKOV-3 cells was determined by real-time PCR. The researchers also found that CR-NiO could enhance the expression of pro-apoptotic genes Bax and caspase-3, and reduce the expression of the anti-apoptotic BCL-2 gene, which proved that CR-NiO is a cheap, stable and more effective drug delivery system against OC.

Curcumin, a fat-soluble polyphenol compound extracted from *Amomum Tsao-Ko Crevostet*, has a wide range of pharmacological activities such as antioxidant, anti-inflammatory and anti-tumor (80). As a natural medicine widely studied in clinical studies, the challenges restricting the clinical application of curcumin mainly come from its low stability and low solubility in solution, as well as poor absorption in the intestines and rapid metabolism after oral administration. Bondì et al. (184) embedded curcumin into SLNs to improve its bioavailability and antitumor activity. In this work, Nanostructured Lipid Carriers (NLCs) loaded with curcumin were prepared using precipitation techniques. By comparing the free curcumin with the NLCs loaded with curcumin, it was found that NLCs loaded with curcumin can induce the decrease of anti-apoptotic proteins such as Bcl-2, Mcl-1 and survivin, activate p38 MAPK to promote apoptosis and down-regulate the homeostasis of IL-6 in OC cells with superior efficacy. Surprisingly, curcumin was also found to inhibit the expression of β-catenin in A2780S cells and A2780CP cells, suggesting that β-catenin is a potential new target for curcumin anti-OC therapy.

#### 4.1.2 SLNs drug delivery system

SLNs are mainly composed of solid lipid molecules such as fatty acids, triglycerides and phospholipids. When assembled with drugs, hydrophobic drugs can be dissolved in its hydrophobic core. With a particle size of 100-200 nm, SLNs can effectively cross the blood-brain barrier, and their unique biocompatible characteristics not only extend the circulation time of drugs in the body but also effectively avoid drug leakage (185). The unique feature is that SLNs can deliver physicochemically incompatible drugs, even those with poor pharmacokinetic profiles (186).

PTX is the most commonly used drug with SLNs used in the treatment of OC, due to early pharmacological studies on PTX and multiple drug delivery systems have been tried by researchers. Tarr et al. found that the solubility of PTX in general lipids is so low that conventional formulations are inadequate as a delivery system for PTX (187). Therefore, to solve the problem, Lee et al. developed a new PTX-loaded sterically stabilized SLN, which uses trimyristin as a lipid solid core and egg phosphatidylcholine and distearoylphosphatidyl-ethanolamine-N-poly-(ethylene glycol)2000 (PEG2000–PE) as a stabilizer changes the characterization of solid lipids in terms of morphology, charge, drug incorporation mode, and stability, making PTX-loaded sterically stabilized SLN a potential alternative to traditional drug delivery strategies (188). The researchers found that the load of PTX increased the average diameter of SLN from 128nm to 217nm, but did not affect the morphology and ideal potential of SLN. Meanwhile, the drug release curve *in vitro* indicated that PTX may bind to SLN through an association relationship and be absorbed into cells by granule administration, playing a slow release role. Tumor cell killing experiments showed that PTX-loaded sterically stabilized SLN showed dose-dependent cytotoxicity to SKOV-3 cells, with 15% higher lethal energy than PTX at the same dose, and the ICso value was about 20uM. In other tumor cells, SLN also acts as a coat to participate in a variety of natural drug conjugates, synergistically participating in targeted drug delivery and inducing tumor cell apoptosis. Kumar et al. evaluated the efficacy of chitosan-coated trans-resveratrol and ferulic acid-supported SLN in targeting colon cancer with FA conjugates. The results showed that natural drugs with SLN as the carrier had a significant anti-tumor effect, significantly inducing apoptosis of tumor cells compared with free drugs (189).

### 4.2 Chitosan-based drug delivery system

Chitosan, the product of alkaline deacetylation of chitin, is the main derivative component of chitin and a natural component of invertebrate exoskeletons. As it has many properties such as hemostasis, promoting wound healing, reducing blood cholesterol levels, and anti-tumor, Chitosan has become a widely used natural drug preparation in the biomedicine field in recent years (190). Since the molecular weight of chitosan is 20-200nm, which conforms to the typical nanostructure characteristics, it has good biodistribution and degradability of nanocarriers. In the treatment of OC, chitosan has been shown not only to inhibit the proliferation of OC cells and induce apoptosis in a dose-dependent manner but also to participate in gene and immunotherapy as a carrier of drug delivery (191). Therefore, compared with conventional carriers, chitosan drug delivery systems have unique advantages in nature, as both the shell and the internal-loaded drugs can play an anti-tumor therapeutic effect, proving to have a multiplier effect with half the effort (192).

DOX, a natural anthracycline derivative derived from the Streptomyces *Peucetius*, has extensive anti-tumor effects on lung cancer, breast cancer, OC, and other tumors (193). Due to the low bioavailability and short half-life of DOX, the key is that effective treatment often requires high drug doses, which often lead to an increase in side effects such as nephrotoxicity and cardiotoxicity. A combination of DOX and other chemotherapy drugs is seen as a promising approach. However, it can only reduce the toxic side effects of DOX, and cannot improve the tumor targeting and drug delivery ability of drug therapy. Using chitosan materials, HU et al. (194) designed and developed a novel acid-sensitive DOX drug delivery system, CMC-CBA-DOX nanoparticles, which passively released DOX in the acidic environment of tumor cells through the covalent bond between Carboxymethyl chitosan(CMC) and DOX. Yan et al. (195) prepared polyvinyl alcohol/chitosan nanofibers with complete biocompatibility for DOX delivery. The experimental results showed that the fiber material could inhibit the proliferation and adhesion of SKOV-3 cells and control the release of DOX, which was also found in a kind of magnetic ferric oxide nanoparticles coated with chitosan (196). Effectively reducing the toxic side effects of drugs is a key issue in the development of NNDDS. Although the toxic side effects of natural drugs are not so strong compared with synthetic drugs, the effect of a series of natural drugs represented by DOX on bone marrow suppression in the body still cannot be ignored. The chitosan nanoparticles developed by Youse et al. (197) have solved this problem well. By using succinic anhydride as a crosslinking agent for the combination of chitosan and DOX, the authors developed nanoparticles with narrow particle size distribution and a clear core-shell structure. Experimental results show that this novel chitosan nanostructure can distinguish between Her 2+ and Her 2- cells, and Her 2+ cells can selectively uptake it, which obviously weakens the cytotoxic effect of DOX on the body and enhances the potential application value of DOX. Meanwhile, the authors of this study also point out the potential application of PH-sensitive copulants in the development of drug delivery systems.

*Camptotheca acuminata Decne.* is a unique Chinese tree plant in the daucrate family, containing the bioactive ingredient, camptothecin (CPT), which has high medical value in pharmaceutical and biomedical fields. CPT possesses a unique 5-fused ring structure, can specifically block the synthesis of topoisomerase I, and has good anticancer activity against OC, gastric cancer, liver cancer and other tumors. However, the pharmacokinetics of CPT are poor and the toxicity of intravenous administration is high, so effectively breaking through the above limitations is the key to expanding clinical application. Li et al. (198) prepared a novel CPT local drug delivery system using chitosan and disodium hydrogen phosphate as carriers. A series of studies, such as degradation experiments, *in vitro* release experiments, and cytotoxicity experiments, have shown that the new drug delivery system is biocompatible and degradable, and the bioactivity of CPT loaded in it can be retained for more than 1 week. Of the drug release days included in the calculation, a total of 70% of CPT was released from the hydrogel within 18 days, and the drug delivery system maintained a good anti-tumor effect even at lower drug concentrations. In order to improve the low water solubility and severe side effects of CPT, Zhou et al. (199) established a safe and effective N-trimethyl chitosan (TMC) coated with the CPT drug delivery system, CPT-TMC. Human ovarian cancer SKOV-3 cells transfected by VEGF-D recombinant plasmid (SKOV-3/VEGF-D) cells were used to construct a lymphoid metastasis model of OC in nude mice to evaluate the anti-tumor and anti-metastatic activity of CPT-TMC and its side effects. The experimental results showed that CPT-TMC could significantly reduce tumor-related blood vessel and lymphangiogenesis, participate in the down-regulation of VEGF-D and MMP-9 expression, increase tumor apoptosis index, maximize the anti-tumor and anti-metastasis activity of CPT, and reduce systemic side effects caused by drugs.

### 4.3 Polymer-based drug delivery system

#### 4.3.1 Polymer nanoparticles

In the past few decades, different types of polymer-based drug delivery systems have been developed, including polymer nanoparticles, polymer micelles, polymer drug couplings, dendritic polymers, etc., providing safer and more effective protocols for the clinical treatment of tumors. Polymer nanoparticles are the most widely used and effective polymer drug delivery system. By packaging and combining hydrophilic polymers of different proportions, polymer nanoparticles can achieve a constant rate of release of therapeutic drugs, of which the surface functional design can give drugs the ability of targeted therapy.

Kaempferol is a natural polyphenol compound, widely found in the daily consumption of vegetables, fruits and some Chinese herbs. Studies have shown that the natural dietary compound Kaempferol exerts anti-inflammatory effects by down-regulating the NF-κB pathway, inhibiting interleukin-4 and cyclo-oxygenase 2 expression (200). In terms of anti-OC treatment, Kaempferol has been reported to reduce the risk of OC risk events (201).

To better improve the bioavailability of Kaempferol, Luo et al. (202) developed five different types of polymer nanoparticles loaded with Kaempferol and evaluated its effect on the cell viability of OC. The results show that compared to other polymer drug delivery systems, Poly(DL-lactic acid-co-glycolic acid) (PLGA) nanoparticles incorporating kaempferol can reduce the vitality of OC cells without altering normal ones, indicating that it had cytoselective toxicity and could be a promising candidate for the prevention and treatment of OC.

Hypericin, a dianthranone compound, is a natural photosensitizer extracted from *Hypericum perforatum L.*, which plays a role in inhibiting tumor cell growth in drug photodynamic therapy against cervical cancer, breast cancer, colon cancer, and liver cancer (203). In Labouebe’s (204) research, polymer nanoparticles based on PLA and PLGA were used as Hypericin drug delivery systems. It was found that free Hypericin is more inclined to passively diffuse into the cell membrane due to its hydrophobic properties, while the drug delivery system, affected by nanoparticle endocytosis, increases the concentration of the drug in the cell. Compared with free Hypericin, Hypericin-loaded PLA Nanoparticles present stronger *in vitro* photoactivity, which can significantly reduce the drug dose of Hypericin. Due to the unique hydrophobicity of Hypericin, it is quite difficult to avoid the poor encapsulation rate of the drug loading system, making it also difficult to prepare this drug loading system. Fortunately, the photoactivity of Hypericin is still maintained after nanoencapsulation. However, the full details of the mechanism of the Hypericin drug delivery system against OC were not disclosed in the original study, making its clinical application uncertain.

Genistein (GEN) is a kind of natural isoflavone existing in Glycine max (L.) Merr., which has the pharmacological effects of regulating steroid hormone receptors, regulating body metabolism, inhibiting inflammation, protecting nerves and anti-tumor (205). The anti-cancer effects of GEN against a variety of cancer types have been studied, but its low water solubility and bioavailability, rapid metabolism and excretion, and lack of specific targeting ability to tumor cells, limiting its clinical application. Arjun et al. (206) developed a GEN-containing FA receptor targeting and pegylated polynanoparticles (PLGA-PEG-FA NPs) that can deliver GEN targeting to OC cells. The study found that PLGA-PEG-FA NPs have the ability to continuously release GEN while increasing the uptake of the drug by SKOV-3 cells, with the potential for better specific delivery compared to non-targeted drugs. The development of this study provides a way for the research and development of GEN targeting nanoparticles, and provides a reference for the co-delivery of imaging agents based on diagnosis in the future.

Curcumin is one of the most widely studied natural drugs in the field of polymer nanoparticles. Sathish et al. (207) encapsulated curcumin in a hydrophilic polymer Poly(2-hydroxyethyl methacrylate) (PHEMA) using nanoprecipitation technology to enhance the anti-tumor targeting ability of curcumin.

The researchers increased the drug-carrying capacity of the drug carrier system to 26.4%. The anti-cancer activity experiment showed that the polymer drug carrier system had better regression activity of ovarian tumor cells than free curcumin, and could exert an anti-OC effect by significantly reducing G_0_/G_1_ phase cells, inhibiting NF-κB function, up-regulating the expression of Bax protein, and down-regulating the expression of Bcl-2 protein, anti-apoptotic survivin, VEGF and COX-2. Another interesting study showed that a polymer-loaded system of curcumin can double the uptake of cisplatin-resistant A2780CP cells and sixfold increase the uptake of metastatic MDA-MB-231 cells. Another interesting study showed that a polymer-loaded system of curcumin respectively increased drug uptake twice for cisplatin-resistant A2780CP cells and six times for metastatic MDA-MB-231 cells, of which the anti-tumor mechanism is related to the enhancement of tumor cell apoptosis induced by Nano-CUR6. The coupling of nanoparticles with anti-cancer antibodies also enables curcumin to achieve tumor-specific targeted delivery (208). *In vivo* experiments also showed that in the experimental group treated with curcumin nanoparticles, the animal ovary histology showed atypical hyperplasia, without tumor inflammatory cells, rough chromatin, and bleeding. Curcumin nanoparticles can inhibit the proliferation of tumor cells by down-regulating the JAK/STAT3 and PI3K/Akt signaling pathways, reducing the expression of JAK and reducing the concentration of TGF-β in ovarian tissue, which significantly affects the phosphorylation process of STAT3 (209). Unfortunately, among the original published studies of polymer drug delivery systems, clinical studies of curcumin against gynecological tumors have been ignored. Even though curcumin has been shown to have extremely high safe doses, its application from the laboratory to any clinical setting must be very cautious.

#### 4.3.2 Polymer micelles

As a nano-delivery system, polymer micelles are self-assembled products of amphiphilic polymers in an aqueous environment, which are widely used to improve the water solubility of chemotherapy drugs in cancer therapy. Polymer micelles are one of the most biocompatible, non-immunogenic, stable and targeted drug delivery carriers with the ability to induce stealth of hydrophilic polymer brush on micelle surface and stable encapsulation capability provided by hydrophobic and rigid micelle core (210). In recent years, it has become a research hotspot that studies and develops ligand modifications, stimulatory responses and multifunctional polymer micelles for tumor drug-targeted therapy, improving micellar blood circulation time and stabilizing drug load (211).

Fisetin is a natural compound extracted from the leaves and stems of the sumac plant and found in a wide range of fruits and vegetables. At high doses, Fisetin has not shown adverse effects, making it an important drug for the study of aging and age-related diseases (212). Studies have shown that Fisetin can play an anti-OC role in various ways such as anti-inflammatory, anti-proliferation and apoptosis-inducing (213). However, the fact that Fisetin has very low water solubility (less than 1mg/g), and unmodified Fisetin has almost no efficacy to play a role, seriously hinders its application in the clinical treatment of OC. Xue prepared polymeric micelles encapsulating fisetin, which greatly improved the physical properties of Fisetin, and the characteristics of micellar carriers also enabled Fisetin to better gather into OC tissue (214). The experimental results showed that Fisetin could down-regulate the expression of Bcl-2 and up-regulate the expression of Bax, briking the balance between Bax and Bcl-2. Apoptosis of SKOV-3 cells was activated by the mitochondrial pathway, and the tumor size and weight were reduced, which effectively inhibited the growth of transplanted tumors in mice. Polymeric micelles encapsulating fisetin also showed a stronger inhibitory effect on tumor growth and prolonged survival compared with free fisetin.

Another interesting study found the ability of co-delivery of resveratrol and curcumin polymer micelles to reduce cardiotoxicity caused by DOX *in vitro*. In the study of Lisa et al. (215), polymer micelles made the water solubility of resveratrol and curcumin 29.6 times and 1617 times higher than their intrinsic solubility. The unique properties of micelles enhanced the retention time of drugs, not only acting as chemical sensitizers for DOX, but also delivering targeted drugs to reduce the occurrence of adverse reaction events. This study demonstrated the potential of co-delivery of two natural compounds in polymer micelles. Compared with conventional micelles, polymer micelles have the advantages of low toxicity, solubilization and structural stability, but the drugs encapsulated in the drug delivery process still have material selectivity. In the study of Sachin K. et al. (216), the researchers encapsulated ceramide with natural sweet tea side and prepared a nanomicelle with high bioavailability, demonstrating for the first time that natural nanomicelles can effectively deliver ceramide *in vivo*. It was found that compared with previous synthetic polymer micelles encapsulated ceramides, the new nanomicelles not only promoted drug delivery to OC cells, but also overcame tissue barriers, inhibited glycosylation, better participating in restoring tumor suppressor activity in OC cells carrying p53 mutations.

### 4.4 A system based on metal nanoparticles

The three major advantages of metal and oxide nanoparticles in medicine are their strong stability, large surface area and good size effect. These advantages are not only reflected in modifying the surface of the nanoparticles to regulate the role they play in the body but also provide multiple surface binding sites for the drug, allowing the drug delivery system to better actively target therapy in the body (217). Gold, a precious and less toxic metal believed to have a calming and detoxifying effect, is also an ingredient in Angong Niuhuang pills in traditional Chinese medicine. In recent years, as gold has extremely high stability, more and more research on nanoparticles based on gold is being carried out.

Wang et al. (93) constructed a drug delivery system gold nanodots-PTX-polylysine (AuNDs-PTX-PLL) based on gold nanoparticles loaded with PTX, which has been proved by experiments to realize intelligent responsive drug delivery, effectively solving the problems of poor water solubility and drug resistance of PTX. In addition, the system reduces damage to normal tissue by intelligently delivering drugs in an acidic tumor environment. The multimodal imaging technology also shows that AuNDs-PTX-PLL nanosystem can also be used to assist tumor diagnosis, achieving accurate diagnosis and treatment of tumor. PTX has a wide range of anti-tumor efficacy against OC, breast cancer and other tumors, although the anti-tumor effect evaluated in the AuNDs-PTX-PLL nanosystem study was carried out on non-small cell lung cancer cell lines, this study also indicates the future research potential of gold nanodrug delivery systems.

Nano-silver, also known as colloidal silver or silver nanoparticles, is an excellent antimicrobial agent. In recent years, more and more studies have been conducted on its effects in organisms and cells, indicating that nano-silver has high research value in the medical field. In a study by Saratale et al. (218), a green silver nanoparticle (Li-AgNPs) prepared from Wheat straw lignin and AgNO3 was shown to be cytotoxic to SKOV-3 cells *in vitro*, suggesting that Li-AgNPs are a promising anti-OC agent.

*Rhynchosia suaveolens (L.f.) DC.* widely distributed in southern India, the leaves are rich in flavonoids and phenolic compounds with anti-cancer and antioxidant activities (219). Another interesting study reports a novel silver nanoparticle drug delivery system (RS-AgNPs) loaded with leaf extract of *Rhynchosia suaveolens (L.f.) DC.* In this drug loading system, loaded with extracts not only plays a major role in anticancer effects, but also reduces silver cations in silver nitrate solution, promoting the formation of bioderived silver nanoparticles during the interaction (220). Studies have confirmed that RS-AgNPs have anticancer activity similar to doxorubicin (DOX), and have targeted anti-proliferation and pro-apoptosis ability against SKOV-3 cells, playing an anticancer role through ROS production and apoptosis signaling pathway switching after DNA fragmentation.

Strictly speaking, this kind of method of reducing metal ions with plant extract to prepare nanoparticles does not belong to NNDDS, because these components only have a reduction reaction with metal ions, effectively remove free radicals, prevent the oxidation chain reaction, and then obtain highly active nanoparticles, which does not involve the category of drug loading. However, it should be noted that the types and components of natural drugs are different, so that the size, shape, and chemical properties of the synthesized nanoparticles are different. In these prepared nanoparticles, we can see the “shadow” of natural products and the properties they “inherit” from natural products, so a simple explanation is made in this part. The detailed anti-OC mechanism of NNDDS is shown in **Table 2**.

**Table 2:** Types of NNDDS and summary of anti-OC mechanisms.

Classification	Natural medicine	Natural sources	Drug delivery system	Cell Line/Animal Model	Mechanism and effect	Ref
EV drug delivery system	Anthocyanin	Blueberries, strawberries and other berries	Anthocyanin Exo preparation	A2780 cells; Athymic nude mice (5-6 weeks old)	Exo synergistically enhances anthocyanin bioavailability and structural stability, and the anthocyanin Exo vector reduces the effective dose of cisplatin required to inhibit cisplatin-resistant ovarian cancer cells and enhances the ability of anthocyanins to inhibit the growth of ovarian cancer cells.	(174)
	Bilberry-derived anthocyanins	*Vaccinium parvifolium Sm.*	Nano preparations of Anthocyanin derived from bilberry	OVCA-432 cells; Wild-type C57BL/6 mice (5-6 weeks old)	Exo improves the stability of Bilberry-derived anthocyanins, causes inhibition of TNFα-induced NF-κB activation in tumor cells, and exerts anti-inflammatory and anti-tumor effects.	(171)
	TP	*Tripterygium wilfordii Hook. f.*	TP-EXOs	SKOV-3 cells, A2780 cells; Balb/c nude mice (4-6 weeks old)	EXO prolongs the time required for TP release and promotes TP recruitment to the tumor site; TP-EXOs can block ovarian cancer cells in the S phase, inhibit cell proliferation and induce apoptosis.	(175)
	PTX	*Taxus wallichiana ZucC.*	MSC-derived PTX-EXOs	SKOV-3 cells; A549 cells; MDA-hyb1 breast cancer cells	MSC-derived PTX-EXOs have better specificity and more effective tumor targeting, which reduces the rate of distant organ metastasis of the tumor and the concentration of PTX during treatment.	(176)
Synthetic Drug Loaded Liposomes	Chrysin	*Passiflora caerulea L., Oroxylum indicum (L.) Kurz and other plant sources*	Chr-NiO	SKOV-3 cells	Chr-NiO significantly reduced the migration ability of SKOV-3 cells, enhanced the expression of pro-apoptotic genes Bax and caspase-3, and reduced the expression of anti-apoptotic BCL-2 genes.	(183)
	Curcumin	*Curcuma longa L.*	NLCs loaded with curcumin	A2780S cells; A2780CP cells	Induces the reduction of anti-apoptotic protein Bcl‐ 2, Mcl-1 and survivin, activates p38 MAPK to initiate pro-apoptosis, down-regulates the steady-state level of cytokine IL-6, and inhibits the expression of β-catenin.	(184)
SLNs drug delivery system	PTX	*Taxus wallichiana ZucC.*	PTX-loaded sterically stabilized SLN	SKOV-3 cells	PTX may bind to SLN through an association relationship, and be absorbed into cells in the form of particles to play a slow-release effect, showing dose-dependent cytotoxicity to SKOV-3 cells.	(188)
Chitosan drug delivery system	DOX	Peucetius Streptomyces	Carboxymethyl chitosan-CBA-DOX nanoparticles	SKOV-3 cells	Improve the control of drug release rate and passive release of DOX in an acidic environment.	(194)
			Polyvinyl alcohol/chitosan nanofibers	SKOV-3 cells	Inhibit the proliferation and adhesion of SKOV-3 cells and control the release of DOX.	(195)
			Chitosan-coated superparamagnetic iron oxide Nanoparticles	A2780 cells; OVCAR-3 cells	It has a high drug loading rate and better performance inhibition of ovarian cancer cell growth.	(196)
			Chitosan-DOX conjugate nanoparticles with anti-Her2 trastuzumab on its surface	Her2^+^ ovarian carcinoma cell line; Her2^−^ human breast cancer cell line	Participate in active targeted drug delivery and inhibit DOX proximity to the target to reduce the cytotoxicity of DOX.	(197)
	CPT	*Camptotheca acuminata*	Chitosan/dibasic sodium phosphate hydrogel	SKOV-3 cells	The drug-loaded system prolongs the activity time of CPT, and achieves a good anti-tumor effect at a lower concentration by sustained release of CPT.	(198)
			CPT-TMC	SKOV-3/VEGF-D cells; Athymic BALB/c nude mice (6-8 weeks old)	CPT-TMC plays a significant role in anti-lymphatic production and anti-angiogenesis, lowering the expression of VEGF-D and MMP-9, reducing LMVD, MVD and KI-67 positive cells, increasing the tumor apoptosis index, and reducing the systemic side effects caused by CPT.	(199)
Polymer nanoparticles drug delivery system	Kaempferol	Widely found in the daily consumption of vegetables, fruits and some Chinese herbal medicine	Kaempferol-PLGA	A2780/CP70 cells; SKOV-3 cells; IOSE 397 cells	Kaempferol-PLGA showed cell selection toxicity, reducing the viability of ovarian cancer cells while not changing the viability of normal cells.	(202)
	Hypericin	*Hypericum monogynum L.*	Hypericin-loaded PLA Nanoparticles	NuTu-19 cells	Compared with free drugs, the Hypericin-loaded PLA Nanoparticles enhance the photoactivity of Hypericin *in vitro*, reduce the negative impact of drug loading on the photoactivity of NuTu-19 cells, and reduce the dose of drug therapy.	(204)
	Genistein	*Glycine max (L.) Merr.*	PLGA-PEG-FA Nanoparticles	SKOV-3 cells	PLGA-PEG-FA Nanoparticles have better hydrophilic properties and better release GEN to exert the cytotoxic effect of targeted drug delivery.	(206)
	Curcumin	*Curcuma longa L.*	Curcumin-PHEMA-Nanoparticles	SKOV-3 cells	PHEMA-based drug delivery system can reduce the number of G_0_/G_1_ phase cells, inhibit the function of NF-κB, down-regulate the expression of Bcl-2 protein, up-regulate the expression of Bax protein, and reduce the expression of anti-apoptotic survivin, VEGF and COX-2.	(207)
			Nano-CUR6	A2780CP cells; MDA-MB-231 cells	Nano-CUR6 shows better anti-tumor activity and tumor-specific targeted delivery ability in cell proliferation and clonal detection. The mechanism of its anti-tumor effect is related to the induced enhanced apoptosis of tumor cells.	(208)
			Nanocurcumin	Female Wistar rats	Nanocurcumin synergistically enhance the anticancer activity of cisplatin by inhibiting TGF-β and IL-6-mediated, down-regulating PI3K/Akt and JAK/STAT3 signaling pathways.	(209)
Polymer micelles drug delivery system	Fisetin	*Anacardiaceae R.Br.*	Polymeric micelles encapsulating fisetin	SKOV-3 cells; female Balb/c nude mice (4-6weeks old)	Polymeric micelles encapsulating fisetin activate Caspase-3 and Casapse-9, down-regulate Bcl-2 expression, up-regulate Bax expression, break the balance between Bax and Bcl-2, and induce SKOV- 3 cell apoptosis through mitochondrial pathway in a dose-dependent manner.	(214)

## 5 Conclusion

**Figure 2:**
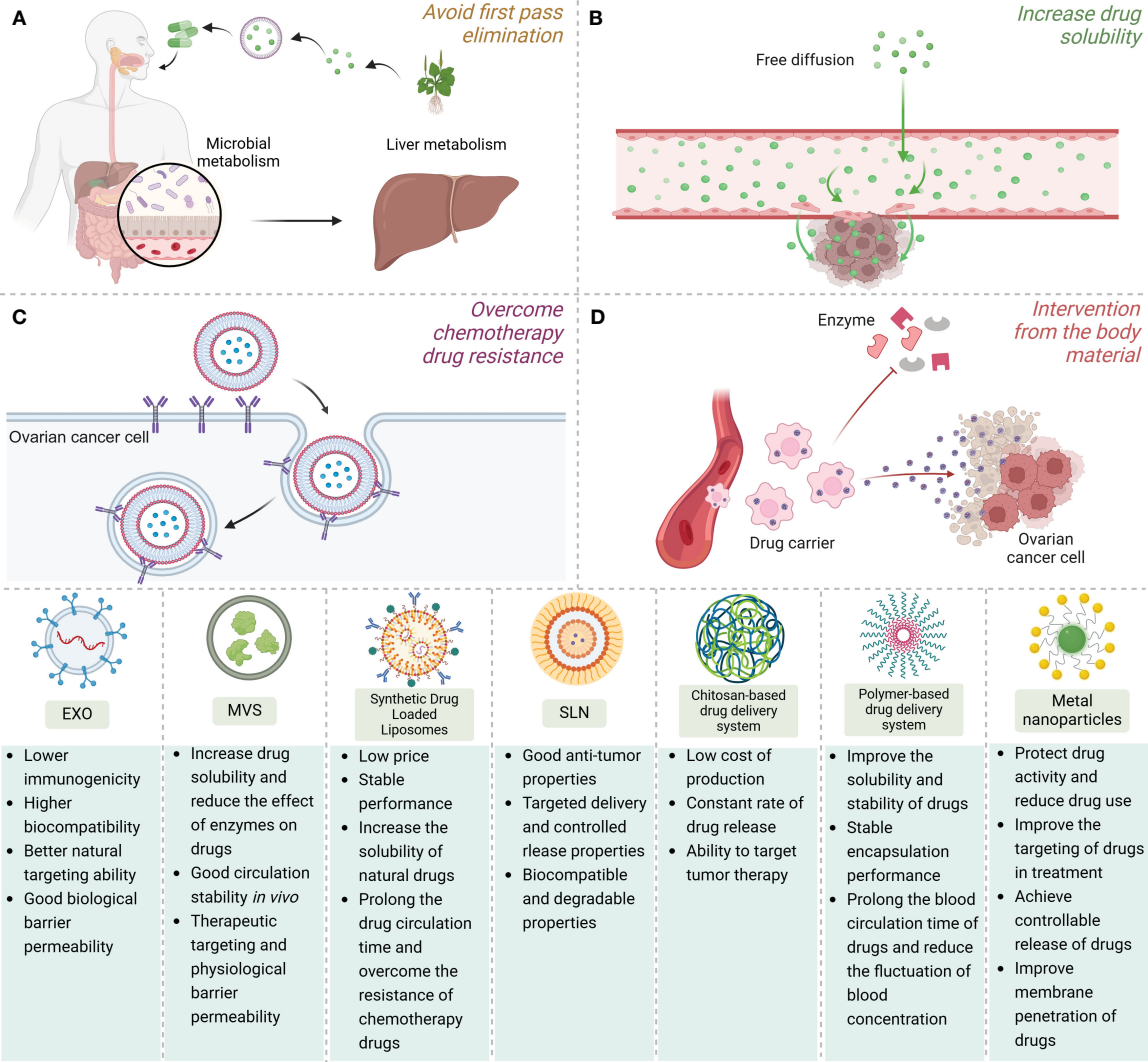
The application advantages, structural simulation demonstration and comparison of advantages and disadvantages of various NNDDS. NNDDS can achieve the selective release of drugs and improve the therapeutic effect by **(A)** avoiding the first-pass clearance, **(B)** increasing the solubility of natural drugs, **(C)** overcoming chemotherapeutic drug resistance through endocytosis, and **(D)** protecting natural drugs from the intervention of substances in the body.

In this manuscript, we conduct a comprehensive summary and discussion of NNDDS targeted for drug delivery against OC. By analyzing its mechanism of action, application implications, and future potential, we reveal the importance and future prospects of this field. Our research not only expands the understanding of the field, but also provides useful guidance for future clinical treatment and drug development. Through in-depth analysis of the mechanism of action of NNDDS in anti-OC therapy, we found that this system can improve the stability and bioavailability of drugs by encapsulating drugs in nanoparticles, alter the pharmacokinetics and pharmacodynamics of drugs, and reduce the toxic side effects of drugs on healthy tissues. This is done primarily by (a) avoiding first-pass clearance increases circulating half-life and prolongs drug circulation time, (b) increasing the solubility of the natural drug, (c) overcoming chemotherapeutic drug resistance through intracellular delivery by endocytosis rather than diffusion, and (d) protecting the natural drug from interference by substances in the body such as various enzymes **Figure 2**. In addition, we also found that NNDDS can achieve selective drug release and improve therapeutic efficacy by targeting OC cell surface receptors and driving specific ligand functions (221).

In order to maximize the anti-tumor potential of natural compounds, it is necessary to improve the efficiency of research and development actions, which is particularly important to focus on direct targets for targeted therapy of cancer. Direct targets of many of the natural compounds in this manuscript have been discovered, greatly promoting the treatment of OC and other malignant tumors. For example, the natural product Anibamine is a chemokine receptor CCR5 antagonist, and then new drug development based on this target has been carried out in ovarian and prostate cancer (64, 222). PCSK9, an enzyme involved in regulating lipid homeostasis by targeting low-density lipoprotein receptor degradation, has been found to play an important role in OC metabolism (223). In addition, Berberine, as a multi-target natural PCSK9 inhibitor, can greatly reduce the cost of drug synthesis (224). Curcumin is a novel p300/CREB binding protein specific acetyltransferase inhibitor and Pan HDAC inhibitor (225, 226). Recent studies have shown that HDAC inhibitors can induce OC sensitivity to cisplatin therapy and reverse the adverse effects of immunotherapy due to loss of homologous recombination defective functional phenotype (227, 228). Involved in numerous cellular processes including metabolism, oxidation, and inflammation, Nrf2 is a transcription factor that regulates different enzymes, receptors and miRNAs (229). Besides, It enhances cellular defense against undesirable stimuli by regulating the expression of related genes. In studies on OC, Nrf2 has been found to play an important regulatory role in the progression, proliferation and chemotherapy resistance of OC. In addition, Nrf2 has been found to regulate the expression of ERα and PGR in OC cells, as a consequence, Nrf2 is regarded as a promising target for future treatment of OC (230). Interestingly, our research has found that Nrf2 is the direct target of many natural compounds such as Carnosol, Resveratrol and Sulforaphane, which is undoubtedly beneficial for future research on the treatment of OC (231–233). Natural compounds, such as Voacamine, an emerging EGFR inhibitor, Withaferin A, a potent inhibitor of the transcription factor C/EBP-β, Quercetin, which suppresses JAK-STAT and telomerase activity, and Oroxylin A, a novel CDK9 and RON protein inhibitor, represent a diverse array of bioactive substances with potential therapeutic applications (234–238).

Many natural compound targets are well known, but the exciting thing is that there is not only one direct target for natural compounds. Dihydroartemisinin is a recognized STAT3 inhibitor and PTGS1 inhibitor (239, 240). Ginsenosides Rc is an activator of SIRT1, SIRT6, and FXR (241–243), and coincidentally, these targets are all linked to OC to some extent (244–246). Among the many natural compounds, these substances that have a direct intervention effect on OC are undoubtedly the first ones we consider to apply to NNDDS, which will save researchers a lot of time and effort in drug screening, enhancing the therapeutic potential of NNDDS against OC.

It can be predicted that NNDDS will become an important breakthrough in the field of cancer treatment. Future research can focus on the following aspects. First, the selection of the carrier of the nano drug delivery system. At present, the drug delivery system represented by PLGA nanoparticles, silica nanoparticles and liposome nanoparticles has been proven to help control the proportion of drug release. The encapsulation of carriers plays a key role in the delivery of natural medicines. The stability, solubility and bioavailability of natural drugs can be improved through rational selection and design of carriers, and the targeted delivery of drugs can be realized.

Second, the stability of the multi-drug co-delivery system. With the deepening of the application of natural drugs in OC, even though natural drugs have the advantage of low tumor resistance compared with synthetic drugs, the long-term application of natural drugs will inevitably make us face the problem of tumor drug resistance. Multi-drug co-delivery is an effective means to reduce the occurrence of drug resistance in tumors, but it requires higher requirements for the complex structure of the vector. It is not only necessary to use new synthetic methods to ensure the high uniformity and high-precision characterization of new materials, but also to consider the collection and production costs of drugs required in the co-delivery system (247, 248). A reasonable solution is to design an NNDDS with multi-target modification and multi-environment response, to realize the encapsulation of more than two response units, however, how to balance the delivery efficiency of the target is also worth considering.

Third, almost all current studies have been conducted in *in vitro* or *in vivo* animal models, and some experiments have shown that non-physiological concentrations of the drug are required to obtain some cellular effects, where the problem is not only the *in vivo* availability, but also the dose-response relationship for the treatment of OC. Limitations of natural product chemistry include low water solubility and limited availability of targeted organs, which may limit their therapeutic use in clinical settings (249–251). Therefore, the future clinical application of NNDDS still has a long way to go. The active ingredients of natural medicine are often low, and the choice of dosage has a direct impact on its therapeutic effect. Different drug doses and components may have differentiated anticancer and protective effects on the female reproductive system, so more effective pharmacodynamic evaluation methods are urgently needed to evaluate the effectiveness of NNDDS in clinical application.

Fourth, the green preparation and economic preparation of NNDDS. In the process of synthesis of some nanoparticles, a lot of chemicals need to be consumed, especially by chemical synthesis methods such as vapor deposition and liquid phase reduction. Therefore, the synthesis of targeted and synergical multi-drug nanocarrier materials using non-toxic and environmentally friendly green synthesis methods is also an important part of future development (252, 253). The nano particle synthesis with plant extract as the end agent and reducing agent can not only help to reduce the preparation cost, and improve the operability and scalability of NNDDS, but also make it safer and more environmentally friendly.

What is worth thinking about is that NNDDS needs a thorough biological evaluation and mechanism research before clinical application to ensure that it can play a normal physiological activity and role when entering cells. Not only drug delivery, but also nanomedicine is trying to find new ways to image, diagnose and identify tumors. It is believed that in the future, NNDDS can be used not only for drug delivery, but also for the implementation of new treatment strategies such as gene therapy and immunotherapy, which will provide new ideas and possibilities for personalized medical treatment and accurate treatment.

## Author contributions

YL: Conceptualization, Writing – original draft. QS: Visualization, Writing – original draft. LF: Writing – original draft. CZ: Data curation, Writing – original draft. XJ: Data curation, Writing – original draft. FL: Data curation, Writing – original draft. BP: Funding acquisition, Supervision, Writing – review & editing.

## Glossary

**Table:**

OC	Ovarian cancer
NNDDS	Nanoscale natural drug delivery system
MDR	Multi-drug resistance
SB	Scutellaria baicalensis
MMPS	Matrix metalloproteinases
CDK	Cytocyclin-dependent kinase
AF	Amentoflavone
SKP2	S-phase kinase protein 2
SKOV-3	Human ovarian cancer SKOV-3 cells line
OVCAR-3	Human ovarian cancer OVCAR-3 cells line
ES-2	Human ovarian cancer ES-2 cells line
A2780	Human ovarian cancer A2780 cells line
OVCA-432	Human ovarian cancer OVCA-432 cells line
TME	The tumor microenvironment
EGCG	Epigallocatechin gallate
HO8910	Human ovarian cancer HO8910 cells line
GSP	Grape seed procyanidin
PTX	Paclitaxel
P-gp	P-glycoprotein
F68-LA	F68-linoleic acid
GA	Gambogic acid
PEG-PLA	Polyethylene glycol and polylactic acid
FA	Folic acid
EV	Extracellular vesicle
EXO	Exosomes
MVS	Microvesicles
TP	Triptolide
TP-EXOs	Triptolide-loaded exosomes delivery system
MSC	Mesenchymal stromal cells
A549 cells	Human lung cancer A549 cells line
SLNs	Solid Lipid Nanoparticles
Chr-NiO	Nano-niosomes containing chrysin
NLCs	Nanostructured Lipid Carriers
MAPK	Mitogen‐activated protein kinase
DOX	Doxorubicin
CMC	Carboxymethyl chitosan
CPT	Camptothecin
SKOV-3/VEGF-D	Human ovarian cancer SKOV-3 cells transfected by VEGF-D recombinant plasmid
TMC	Trimethyl chitosan
NuTu-19	NuTu-19 cell line Rat ovarian cancer cell line
PHEMA	Poly(2-hydroxyethyl methacrylate)
GEN	Genistein
EMT	Epithelial to Mesenchymal Transition
ROS	Reactive oxygen species
CHK2	Checkpoint kinase 2
HK2	Hexokinase-2
HIF-1α	Hypoxia-inducible factor 1α
CTR1	Copper transporter 1
YB-1	Y-box binding protein 1
ROS	Reactive oxygen species
IL-8	Interleukin-8
ER-α	Estrogen receptor alpha
PI3K	Phosphatidylinositol 3-Kinase
PPARγ	Peroxisome proliferator−activated receptor gamma
PGRMC	Progesterone receptor membrane component
JNK	c-Jun N-terminal kinase
CCR5	CC chemokine receptor 5
CCL5	CC chemokine ligand 5
AMPK	Adenosine 5’-monophosphate (AMP)-activated protein kinase
mTOR	mammalian target of rapamycin
FBXW7	F-box and WD-40 domain protein 7
Bax	Bcl-2-associated X protein
NF-κB	nuclear factor kappa-B
VEGF	Vascular endothelial growth factor
YAP	Yes-associated protein
RASSF6	Ras association domain family member 6
PCNA	Proliferating cell nuclear antigen
RIPK3	Receptor interacting serine/threonine kinase 3
MLKL	Mixed Lineage Kinase Domain Like Pseudokinase
EGFR	Epidermal Growth Factor Receptor
IL-6	Interleukin-6
STAT3	Signal transducer and activator of transcription 3
BCL2	BCL2 apoptosis regulator
FOXO1	Forkhead box O1
FAK	Focal adhesion kinase
SMG1	SMG1 Nonsense Mediated MRNA Decay Associated PI3K Related Kinase
NRF2	nuclear factor-erythroid 2-related factor 2
ET-1	Endothelin-1
ETBR	Endothelin B receptor
CDK4	Cyclin dependent kinase 4
Bcl-X	BCL2 like 1
AQP5	Aquaporin 5
FOXO3A	Forkhead box O3
ILK	Integrin linked kinase
GSK-3β	Glycogen synthase kinase 3 beta
Slug	Snail Family Transcriptional Repressor 2
ERK	Extracellular regulated protein kinases
KIF20A	Kinesin family member 20A
CDC25A	Cell Division Cycle 25A
PARP	Poly ADP-ribose polymerase
LC3	Microtubule-associated protein 1 light chain 3
PGRMC	Progesterone receptor membrane component
CREB	CAMP responsive element binding protein
AP-1	Activator protein-1
TRAIL	Tumor necrosis factor-related apoptosis-inducing ligand
DR5	Death receptor 5
MMP9	Matrix metallopeptidase 9
EZH2	Enhancer of zeste 2 polycomb repressive complex 2 subunit
BID	BH3 Interacting Domain Death Agonist
ER-α	Estrogen receptor alpha
JAK	Janus kinase
STAT	Signal transducer and activator
Atg5	Autophagy Related 5
Atg7	Autophagy related 7
RAD51	RAD51 recombinase
HK2	Hexokinase 2
TET3	Tet methylcytosine dioxygenase 3
MEG3	Maternally expressed 3
SNIP1	Smad nuclear interacting protein 1
BRCA1	BRCA1 DNA Repair Associated
SLC7A6	Solute carrier family 7 member 6
PDCD4	Programmed cell death 4
PTEN	Phosphatase and tensin homolog
FAK	Focal adhesion kinase
GLI1	GLI family zinc finger 1
BMI1	BMI1 proto-oncogene, polycomb ring finger
YB-1	Y box binding protein 1
Ctr1	Copper transport protein
hTERT	Human telomerase reverse transcriptase
MDR1	Multidrug resistance protein 1
PRAP	poly ADP-ribose polymerase
Cyto c	cytochrome c
AIF	Apoptosis inducing factor

## References

[1] Sung H Ferlay J Siegel RL Laversanne M Soerjomataram I Jemal A. Global cancer statistics 2020: GLOBOCAN estimates of incidence and mortality worldwide for 36 cancers in 185 countries. CA Cancer J Clin. (2021) 71:209–49. doi: 10.3322/caac.21660 33538338

[2] Carrera PM Kantarjian HM Blinder VS. The financial burden and distress of patients with cancer: Understanding and stepping-up action on the financial toxicity of cancer treatment. CA Cancer J Clin. (2018) 68:153–65. doi: 10.3322/caac.21443 PMC6652174 29338071

[3] Jiang Y Wang C Zhou S. Targeting tumor microenvironment in ovarian cancer: Premise and promise. Biochim Biophys Acta Rev Cancer. (2020) 1873:188361. doi: 10.1016/j.bbcan.2020.188361 32234508

[4] Xie H Wang W Xia B Jin W Lou G. Therapeutic applications of PARP inhibitors in ovarian cancer. BioMed Pharmacother. (2020) 127:110204. doi: 10.1016/j.biopha.2020.110204 32422564

[5] Francica P Rottenberg S. Mechanisms of PARP inhibitor resistance in cancer and insights into the DNA damage response. Genome Med. (2018) 10:101. doi: 10.1186/s13073-018-0612-8 30593284 PMC6309079

[6] Newman DJ Cragg GM. Natural products as sources of new drugs over the nearly four decades from 01/1981 to 09/2019. J Nat Prod. (2020) 83:770–803. doi: 10.1021/acs.jnatprod.9b01285 32162523

[7] Gandhi GR Antony PJ Lana M da Silva BFX Oliveira RV Jothi G. Natural products modulating interleukins and other inflammatory mediators in tumor-bearing animals: A systematic review. Phytomedicine. (2022) 100:154038. doi: 10.1016/j.phymed.2022.154038 35358934

[8] Jayakodi S Senthilnathan R Swaminathan A Shanmugam VK Shanmugam R Krishnan A. Bio-inspired nanoparticles mediated from plant extract biomolecules and their therapeutic application in cardiovascular diseases: A review. Int J Biol Macromol. (2023) 242:125025. doi: 10.1016/j.ijbiomac.2023.125025 37245774

[9] Cragg GM Pezzuto JM. Natural products as a vital source for the discovery of cancer chemotherapeutic and chemopreventive agents. Med Princ Pract. (2016) 25 Suppl 2:41–59. doi: 10.1159/000443404 26679767 PMC5588531

[10] Tang P Shen T Wang H Zhang R Zhang X Li X. Challenges and opportunities for improving the druggability of natural product: Why need drug delivery system? BioMed Pharmacother. (2023) 164:114955. doi: 10.1016/j.biopha.2023.114955 37269810

[11] Wang Z Meng F Zhong Z. Emerging targeted drug delivery strategies toward ovarian cancer. Adv Drug Delivery Rev. (2021) 178:113969. doi: 10.1016/j.addr.2021.113969 34509574

[12] Wicki A Witzigmann D Balasubramanian V Huwyler J. Nanomedicine in cancer therapy: challenges, opportunities, and clinical applications. J Control Release. (2015) 200:138–57. doi: 10.1016/j.jconrel.2014.12.030 25545217

[13] Patra JK Das G Fraceto LF Campos EVR Rodriguez-Torres MDP Acosta-Torres LS. Nano based drug delivery systems: recent developments and future prospects. J Nanobiotechnology. (2018) 16:71. doi: 10.1186/s12951-018-0392-8 30231877 PMC6145203

[14] Joseph RR Venkatraman SS. Drug delivery to the eye: what benefits do nanocarriers offer? Nanomedicine (Lond). (2017) 12:683–702. doi: 10.2217/nnm-2016-0379 28186436

[15] Zahin N Anwar R Tewari D Kabir MT Sajid A Mathew B. Nanoparticles and its biomedical applications in health and diseases: special focus on drug delivery. Environ Sci pollut Res Int. (2020) 27:19151–68. doi: 10.1007/s11356-019-05211-0 31079299

[16] Duan L Li X Ji R Hao Z Kong M Wen X. Nanoparticle-based drug delivery systems: an inspiring therapeutic strategy for neurodegenerative diseases. Polymers (Basel). (2023) 15:2196. doi: 10.3390/polym15092196 37177342 PMC10181407

[17] Alqosaibi AI. Nanocarriers for anticancer drugs: Challenges and perspectives. Saudi J Biol Sci. (2022) 29:103298. doi: 10.1016/j.sjbs.2022.103298 35645591 PMC9130109

[18] Michlewska S Garaiova Z Subjakova V Holota M Kubczak M Grodzicka M. Lipid-coated ruthenium dendrimer conjugated with doxorubicin in anti-cancer drug delivery: Introducing protocols. Colloids Surf B Biointerfaces. (2023) 227:113371. doi: 10.1016/j.colsurfb.2023.113371 37244201

[19] Chen H Huang S Wang H Chen X Zhang H Xu Y. Preparation and characterization of paclitaxel palmitate albumin nanoparticles with high loading efficacy: an in *vitro* and in *vivo* anti-tumor study in mouse models. Drug Deliv. (2021) 28:1067–79. doi: 10.1080/10717544.2021.1921078 PMC8205042 34109887

[20] Parayath NN Stephan SB Koehne AL Nelson PS Stephan MT. *In vitro*-transcribed antigen receptor mRNA nanocarriers for transient expression in circulating T cells in vivo. Nat Commun. (2020) 11:6080. doi: 10.1038/s41467-020-19486-2 33247092 PMC7695830

[21] Vergallo C Hafeez MN Iannotta D Santos HA D’Avanzo N Dini L. Conventional nanosized drug delivery systems for cancer applications. Adv Exp Med Biol. (2021) 1295:3–27. doi: 10.1007/978-3-030-58174-9_1 33543453

[22] Dancey J. Targeted therapies and clinical trials in ovarian cancer. Ann Oncol. (2013) 24 Suppl 10:x59–63. doi: 10.1093/annonc/mdt473 24265407

[23] Ottevanger PB. Ovarian cancer stem cells more questions than answers. Semin Cancer Biol. (2017) 44:67–71. doi: 10.1016/j.semcancer.2017.04.009 28450177

[24] Zhang CL Jiang XC Li Y Pan X Gao MQ Chen Y. Independent predictive value of blood inflammatory composite markers in ovarian cancer: recent clinical evidence and perspective focusing on NLR and PLR. J Ovarian Res. (2023) 16:36. doi: 10.1186/s13048-023-01116-2 36759864 PMC9912515

[25] Li Y Zhang C Feng L Shen Q Liu F Jiang X. Application of natural polysaccharides and their novel dosage forms in gynecological cancers: therapeutic implications from the diversity potential of natural compounds. Front Pharmacol. (2023) 14:1195104. doi: 10.3389/fphar.2023.1195104 37383719 PMC10293794

[26] Jiao R Liu Y Gao H Xiao J So KF. The anti-oxidant and antitumor properties of plant polysaccharides. Am J Chin Med. (2016) 44:463–88. doi: 10.1142/S0192415X16500269 27109156

[27] Atanasov AG Zotchev SB Dirsch VM International Natural Product Sciences T Supuran CT. Natural products in drug discovery: advances and opportunities. Nat Rev Drug Discovery. (2021) 20:200–16. doi: 10.1038/s41573-020-00114-z PMC7841765 33510482

[28] Yang F Yu XH Qiao F Cheng LH Chen G Long X. Formulation and characterization of Brucea javanica oil microemulsion for improving safety. Drug Dev Ind Pharm. (2014) 40:266–77. doi: 10.3109/03639045.2012.756887 23356859

[29] Xie C Zhan T Huang J Lan J Shen L Wang H. Functional characterization of nine critical genes encoding rate-limiting enzymes in the flavonoid biosynthesis of the medicinal herb Grona styracifolia. BMC Plant Biol. (2023) 23:299. doi: 10.1186/s12870-023-04290-z 37268882 PMC10239141

[30] Middleton E Jr. Kandaswami C Theoharides TC. The effects of plant flavonoids on mammalian cells: implications for inflammation, heart disease, and cancer. Pharmacol Rev. (2000) 52:673–751. 11121513

[31] Rioux LE Turgeon SL Beaulieu M. Structural characterization of laminaran and galactofucan extracted from the brown seaweed Saccharina longicruris. Phytochemistry. (2010) 71:1586–95. doi: 10.1016/j.phytochem.2010.05.021 20599236

[32] Tzianabos AO. Polysaccharide immunomodulators as therapeutic agents: structural aspects and biologic function. Clin Microbiol Rev. (2000) 13:523–33. doi: 10.1128/CMR.13.4.523 PMC88946 11023954

[33] Garg P Awasthi S Horne D Salgia R Singhal SS. The innate effects of plant secondary metabolites in preclusion of gynecologic cancers: Inflammatory response and therapeutic action. Biochim Biophys Acta Rev Cancer. (2023) 1878:188929. doi: 10.1016/j.bbcan.2023.188929 37286146

[34] Mize BK Salvi A Ren Y Burdette JE Fuchs JR. Discovery and development of botanical natural products and their analogues as therapeutics for ovarian cancer. Nat Prod Rep. (2023) 40:1250–70. doi: 10.1039/D2NP00091A PMC10448539 37387219

[35] Vergara D De Domenico S Tinelli A Stanca E Del Mercato LL Giudetti AM. Anticancer effects of novel resveratrol analogues on human ovarian cancer cells. Mol Biosyst. (2017) 13:1131–41. doi: 10.1039/C7MB00128B 28429008

[36] Feng J Zhang XL Li YY Cui YY Chen YH. Pinus massoniana bark extract: structure-activity relationship and biomedical potentials. Am J Chin Med. (2016) 44:1559–77. doi: 10.1142/S0192415X16500877 27852122

[37] Nan Y Su H Zhou B Liu S. The function of natural compounds in important anticancer mechanisms. Front Oncol. (2022) 12:1049888. doi: 10.3389/fonc.2022.1049888 36686745 PMC9846506

[38] Zhao Y Tan Y Wu G Liu L Wang Y Luo Y. Berbamine overcomes imatinib-induced neutropenia and permits cytogenetic responses in Chinese patients with chronic-phase chronic myeloid leukemia. Int J Hematol. (2011) 94:156–62. doi: 10.1007/s12185-011-0887-7 21728004

[39] Zhang H Jiao Y Shi C Song X Chang Y Ren Y. Berbamine suppresses cell proliferation and promotes apoptosis in ovarian cancer partially via the inhibition of Wnt/beta-catenin signaling. Acta Biochim Biophys Sin (Shanghai). (2018) 50:532–9. doi: 10.1093/abbs/gmy036 29701777

[40] Yan H Xin S Wang H Ma J Zhang H Wei H. Baicalein inhibits MMP-2 expression in human ovarian cancer cells by suppressing the p38 MAPK-dependent NF-kappaB signaling pathway. Anticancer Drugs. (2015) 26:649–56. doi: 10.1097/CAD.0000000000000230 25811965

[41] Lang X Chen Z Yang X Yan Q Xu M Liu W. Scutellarein induces apoptosis and inhibits proliferation, migration, and invasion in ovarian cancer via inhibition of EZH2/FOXO1 signaling. J Biochem Mol Toxicol. (2021) 35:e22870. doi: 10.1002/jbt.22870 34350670

[42] Milde-Langosch K Riethdorf S. Role of cell-cycle regulatory proteins in gynecological cancer. J Cell Physiol. (2003) 196:224–44. doi: 10.1002/jcp.10286 12811815

[43] Liu H Yue Q He S. Amentoflavone suppresses tumor growth in ovarian cancer by modulating Skp2. Life Sci. (2017) 189:96–105. doi: 10.1016/j.lfs.2017.09.026 28942285

[44] Guo Y Zhang Z Wang Z Liu G Liu Y Wang H. Astragalus polysaccharides inhibit ovarian cancer cell growth via microRNA-27a/FBXW7 signaling pathway. Biosci Rep. (2020) 40:BSR20193396. doi: 10.1042/BSR20193396 32159214 PMC7103584

[45] Lee K Ahn JH Lee KT Jang DS Choi JH. Deoxyschizandrin, isolated from schisandra berries, induces cell cycle arrest in ovarian cancer cells and inhibits the protumoural activation of tumour-associated macrophages. Nutrients. (2018) 10:91. doi: 10.3390/nu10010091 29342940 PMC5793319

[46] Cheng X Ferrell JE Jr. Apoptosis propagates through the cytoplasm as trigger waves. Science. (2018) 361:607–12. doi: 10.1126/science.aah4065 PMC6263143 30093599

[47] Xu X Shi J Gao H Li Q. Zeylenone inhibits proliferation and promotes apoptosis in ovarian carcinoma cells via Janus kinase 2/signal transducers and activators of transcription 3 pathways. J Obstet Gynaecol Res. (2018) 44:1451–7. doi: 10.1111/jog.13690 29974554

[48] Zhang L Huo X Liao Y Yang F Gao L Cao L. Zeylenone, a naturally occurring cyclohexene oxide, inhibits proliferation and induces apoptosis in cervical carcinoma cells via PI3K/AKT/mTOR and MAPK/ERK pathways. Sci Rep. (2017) 7:1669. doi: 10.1038/s41598-017-01804-2 28490807 PMC5431878

[49] Che X Yan H Sun H Dongol S Wang Y Lv Q. Grifolin induces autophagic cell death by inhibiting the Akt/mTOR/S6K pathway in human ovarian cancer cells. Oncol Rep. (2016) 36:1041–7. doi: 10.3892/or.2016.4840 27277722

[50] Luo X Xu J Yu J Yi P. Shaping immune responses in the tumor microenvironment of ovarian cancer. Front Immunol. (2021) 12:692360. doi: 10.3389/fimmu.2021.692360 34248988 PMC8261131

[51] Brockmueller A Sameri S Liskova A Zhai K Varghese E Samuel SM. Resveratrol’s anti-cancer effects through the modulation of tumor glucose metabolism. Cancers (Basel). (2021) 13:188. doi: 10.3390/cancers13020188 33430318 PMC7825813

[52] Zhang Z Zhang S Yang J Yi P Xu P Yi M. Integrated transcriptomic and metabolomic analyses to characterize the anti-cancer effects of (-)-epigallocatechin-3-gallate in human colon cancer cells. Toxicol Appl Pharmacol. (2020) 401:115100. doi: 10.1016/j.taap.2020.115100 32512070

[53] Gan RY Li HB Sui ZQ Corke H. Absorption, metabolism, anti-cancer effect and molecular targets of epigallocatechin gallate (EGCG): An updated review. Crit Rev Food Sci Nutr. (2018) 58:924–41. doi: 10.1080/10408398.2016.1231168 27645804

[54] Huang TT Lampert EJ Coots C Lee JM. Targeting the PI3K pathway and DNA damage response as a therapeutic strategy in ovarian cancer. Cancer Treat Rev. (2020) 86:102021. doi: 10.1016/j.ctrv.2020.102021 32311593 PMC7272282

[55] Achi IT Sarbadhikary P George BP Abrahamse H. Multi-target potential of berberine as an antineoplastic and antimetastatic agent: A special focus on lung cancer treatment. Cells. (2022) 11:3433. doi: 10.3390/cells11213433 36359829 PMC9655513

[56] Liu Q Tang J Chen S Hu S Shen C Xiang J. Berberine for gastric cancer prevention and treatment: Multi-step actions on the Correa’s cascade underlie its therapeutic effects. Pharmacol Res. (2022) 184:106440. doi: 10.1016/j.phrs.2022.106440 36108874

[57] Sun Q Yang H Liu M Ren S Zhao H Ming T. Berberine suppresses colorectal cancer by regulation of Hedgehog signaling pathway activity and gut microbiota. Phytomedicine. (2022) 103:154227. doi: 10.1016/j.phymed.2022.154227 35679795

[58] Hou D Xu G Zhang C Li B Qin J Hao X. Berberine induces oxidative DNA damage and impairs homologous recombination repair in ovarian cancer cells to confer increased sensitivity to PARP inhibition. Cell Death Dis. (2017) 8:e3070. doi: 10.1038/cddis.2017.471 28981112 PMC5680592

[59] Disis ML Taylor MH Kelly K Beck JT Gordon M Moore KM. Efficacy and safety of avelumab for patients with recurrent or refractory ovarian cancer: phase 1b results from the JAVELIN solid tumor trial. JAMA Oncol. (2019) 5:393–401. doi: 10.1001/jamaoncol.2018.6258 30676622 PMC6439837

[60] Wu J Zhou T Wang Y Jiang Y Wang Y. Mechanisms and advances in anti-ovarian cancer with natural plants component. Molecules. (2021) 26:5949. doi: 10.3390/molecules26195949 34641493 PMC8512305

[61] Holzapfel NP Shokoohmand A Wagner F Landgraf M Champ S Holzapfel BM. Lycopene reduces ovarian tumor growth and intraperitoneal metastatic load. Am J Cancer Res. (2017) 7:1322–36. PMC5489781 28670494

[62] Milani A Basirnejad M Shahbazi S Bolhassani A. Carotenoids: biochemistry, pharmacology and treatment. Br J Pharmacol. (2017) 174:1290–324. doi: 10.1111/bph.13625 PMC5429337 27638711

[63] Zhao BX Sun YB Wang SQ Duan L Huo QL Ren F. Grape seed procyanidin reversal of p-glycoprotein associated multi-drug resistance via down-regulation of NF-kappaB and MAPK/ERK mediated YB-1 activity in A2780/T cells. PloS One. (2013) 8:e71071. doi: 10.1371/journal.pone.0071071 23967153 PMC3744527

[64] Zhang Y Arnatt CK Zhang F Wang J Haney KM Fang X. The potential role of anibamine, a natural product CCR5 antagonist, and its analogues as leads toward development of anti-ovarian cancer agents. Bioorg Med Chem Lett. (2012) 22:5093–7. doi: 10.1016/j.bmcl.2012.05.127 22770928

[65] Ren L Cao QX Zhai FR Yang SQ Zhang HX. Asiatic acid exerts anticancer potential in human ovarian cancer cells via suppression of PI3K/Akt/mTOR signalling. Pharm Biol. (2016) 54:2377–82. doi: 10.3109/13880209.2016.1156709 26984021

[66] Chen J Li Z Chen AY Ye X Luo H Rankin GO. Inhibitory effect of baicalin and baicalein on ovarian cancer cells. Int J Mol Sci. (2013) 14:6012–25. doi: 10.3390/ijms14036012 PMC3634505 23502466

[67] He Z Li B Rankin GO Rojanasakul Y Chen YC. Selecting bioactive phenolic compounds as potential agents to inhibit proliferation and VEGF expression in human ovarian cancer cells. Oncol Lett. (2015) 9:1444–50. doi: 10.3892/ol.2014.2818 PMC4314987 25663929

[68] Li Y Wang D Liu J Li Y Chen D Zhou L. Baicalin attenuates YAP activity to suppress ovarian cancer stemness. Onco Targets Ther. (2020) 13:7151–63. doi: 10.2147/OTT.S254607 PMC7386807 32801747

[69] Liu L Fan J Ai G Liu J Luo N Li C. Berberine in combination with cisplatin induces necroptosis and apoptosis in ovarian cancer cells. Biol Res. (2019) 52:37. doi: 10.1186/s40659-019-0243-6 31319879 PMC6637630

[70] Li J Zhang S Wu L Pei M Jiang Y. Berberine inhibited metastasis through miR-145/MMP16 axis in vitro. J Ovarian Res. (2021) 14:4. doi: 10.1186/s13048-020-00752-2 33407764 PMC7789793

[71] Chuang TC Wu K Lin YY Kuo HP Kao MC Wang V. Dual down-regulation of EGFR and ErbB2 by berberine contributes to suppression of migration and invasion of human ovarian cancer cells. Environ Toxicol. (2021) 36:737–47. doi: 10.1002/tox.23076 33325633

[72] Yu Z Wan Y Liu Y Yang J Li L Zhang W. Curcumin induced apoptosis via PI3K/Akt-signalling pathways in SKOV3 cells. Pharm Biol. (2016) 54:2026–32. doi: 10.3109/13880209.2016.1139601 26911246

[73] Watson JL Greenshields A Hill R Hilchie A Lee PW Giacomantonio CA. Curcumin-induced apoptosis in ovarian carcinoma cells is p53-independent and involves p38 mitogen-activated protein kinase activation and downregulation of Bcl-2 and survivin expression and Akt signaling. Mol Carcinog. (2010) 49:13–24. doi: 10.1002/mc.20571 19676105

[74] Saydmohammed M Joseph D Syed V. Curcumin suppresses constitutive activation of STAT-3 by up-regulating protein inhibitor of activated STAT-3 (PIAS-3) in ovarian and endometrial cancer cells. J Cell Biochem. (2010) 110:447–56. doi: 10.1002/jcb.22558 20235152

[75] Seo JA Kim B Dhanasekaran DN Tsang BK Song YS. Curcumin induces apoptosis by inhibiting sarco/endoplasmic reticulum Ca2+ ATPase activity in ovarian cancer cells. Cancer Lett. (2016) 371:30–7. doi: 10.1016/j.canlet.2015.11.021 26607901

[76] Zhao SF Zhang X Zhang XJ Shi XQ Yu ZJ Kan QC. Induction of microRNA-9 mediates cytotoxicity of curcumin against SKOV3 ovarian cancer cells. Asian Pac J Cancer Prev. (2014) 15:3363–8. doi: 10.7314/APJCP.2014.15.8.3363 24870723

[77] Sun S Fang H. Curcumin inhibits ovarian cancer progression by regulating circ-PLEKHM3/miR-320a/SMG1 axis. J Ovarian Res. (2021) 14:158. doi: 10.1186/s13048-021-00916-8 34784955 PMC8594156

[78] Barinda AJ Arozal W Sandhiutami NMD Louisa M Arfian N Sandora N. Curcumin Prevents Epithelial-to Mesenchymal Transition-Mediated Ovarian Cancer Progression through NRF2/ETBR/ET-1 Axis and Preserves Mitochondria Biogenesis in Kidney after Cisplatin Administration. Adv Pharm Bull. (2022) 12:128–41. doi: 10.34172/apb.2022.014 PMC9012927 35517894

[79] Liu J Liu X Ma W Kou W Li C Zhao J. Anticancer activity of cucurbitacin-A in ovarian cancer cell line SKOV3 involves cell cycle arrest, apoptosis and inhibition of mTOR/PI3K/Akt signaling pathway. J BUON. (2018) 23:124–8. 29552771

[80] Vergara D Simeone P Bettini S Tinelli A Valli L Storelli C. Antitumor activity of the dietary diterpene carnosol against a panel of human cancer cell lines. Food Funct. (2014) 5:1261–9. doi: 10.1039/c4fo00023d 24733049

[81] Liu Y Gao S Zhu J Zheng Y Zhang H Sun H. Dihydroartemisinin induces apoptosis and inhibits proliferation, migration, and invasion in epithelial ovarian cancer via inhibition of the hedgehog signaling pathway. Cancer Med. (2018) 7:5704–15. doi: 10.1002/cam4.1827 PMC6247066 30338663

[82] Huh SW Bae SM Kim YW Lee JM Namkoong SE Lee IP. Anticancer effects of (-)-epigallocatechin-3-gallate on ovarian carcinoma cell lines. Gynecol Oncol. (2004) 94:760–8. doi: 10.1016/j.ygyno.2004.05.031 15350370

[83] Yan C Yang J Shen L Chen X. Inhibitory effect of Epigallocatechin gallate on ovarian cancer cell proliferation associated with aquaporin 5 expression. Arch Gynecol Obstet. (2012) 285:459–67. doi: 10.1007/s00404-011-1942-6 21698451

[84] Zhang Z Zhang Q Yu Y Su S. Epigallocatechin gallate inhibits ovarian cancer cell growth and induces cell apoptosis via activation of FOXO3A. In Vitro Cell Dev Biol Anim. (2023) 59:739–46. doi: 10.1007/s11626-023-00830-x 38038884

[85] Qin J Fu M Wang J Huang F Liu H Huangfu M. PTEN/AKT/mTOR signaling mediates anticancer effects of epigallocatechin−3−gallate in ovarian cancer. Oncol Rep. (2020) 43:1885–96. doi: 10.3892/or.2020.7571 PMC7160558 32236585

[86] Lu J Xu Y Wei X Zhao Z Xue J Liu P. Emodin inhibits the epithelial to mesenchymal transition of epithelial ovarian cancer cells via ILK/GSK-3beta/slug signaling pathway. BioMed Res Int. (2016) 2016:6253280. doi: 10.1155/2016/6253280 28097141 PMC5206434

[87] Park S Bazer FW Lim W Song G. The O-methylated isoflavone, formononetin, inhibits human ovarian cancer cell proliferation by sub G0/G1 cell phase arrest through PI3K/AKT and ERK1/2 inactivation. J Cell Biochem. (2018) 119:7377–87. doi: 10.1002/jcb.27041 29761845

[88] Gao L Ouyang Y Li R Zhang X Gao X Lin S. Icaritin inhibits migration and invasion of human ovarian cancer cells via the Akt/mTOR signaling pathway. Front Oncol. (2022) 12:843489. doi: 10.3389/fonc.2022.843489 35433438 PMC9010825

[89] Liu T Zhao L Zhang Y Chen W Liu D Hou H. Ginsenoside 20(S)-Rg3 targets HIF-1alpha to block hypoxia-induced epithelial-mesenchymal transition in ovarian cancer cells. PloS One. (2014) 9:e103887. doi: 10.1371/journal.pone.0103887 25197976 PMC4157750

[90] Liu T Zhao L Hou H Ding L Chen W Li X. Ginsenoside 20(S)-Rg3 suppresses ovarian cancer migration via hypoxia-inducible factor 1 alpha and nuclear factor-kappa B signals. Tumour Biol. (2017) 39:1010428317692225. doi: 10.1177/1010428317692225 28459376

[91] Liu D Liu T Teng Y Chen W Zhao L Li X. Ginsenoside Rb1 inhibits hypoxia-induced epithelial-mesenchymal transition in ovarian cancer cells by regulating microRNA-25. Exp Ther Med. (2017) 14:2895–902. doi: 10.3892/etm.2017.4889 PMC5590044 28928801

[92] Zhao L Sun W Zheng A Zhang Y Fang C Zhang P. Ginsenoside Rg3 suppresses ovarian cancer cell proliferation and invasion by inhibiting the expression of lncRNA H19. Acta Biochim Pol. (2021) 68:575–82. doi: 10.18388/abp.2020_5343 34038042

[93] Zhang R Li L Li H Bai H Suo Y Cui J. Ginsenoside 20(S)-Rg3 reduces KIF20A expression and promotes CDC25A proteasomal degradation in epithelial ovarian cancer. J Ginseng Res. (2024) 48:40–51. doi: 10.1016/j.jgr.2023.06.008 38223825 PMC10785255

[94] Chen HY Huang TC Shieh TM Wu CH Lin LC Hsia SM. Isoliquiritigenin induces autophagy and inhibits ovarian cancer cell growth. Int J Mol Sci. (2017) 18:2025. doi: 10.3390/ijms18102025 28934130 PMC5666707

[95] Shen JJ Zhu XF Xu J Wang ZF Gu WJ Chen Y. Oroxylin A exerts anticancer effects on human ovarian cancer cells via the PPARgamma−dependent reversal of the progesterone receptor membrane component 1/2 expression profile. Oncol Rep. (2020) 43:1309–18. doi: 10.3892/or.2020.7509 32323796

[96] Gao J Zhu H Wan H Zou X Ma X Gao G. Harmine suppresses the proliferation and migration of human ovarian cancer cells through inhibiting ERK/CREB pathway. Oncol Rep. (2017) 38:2927–34. doi: 10.3892/or.2017.5952 28901502

[97] Jeong M Kim HM Kim HJ Choi JH Jang DS. Kudsuphilactone B, a nortriterpenoid isolated from Schisandra chinensis fruit, induces caspase-dependent apoptosis in human ovarian cancer A2780 cells. Arch Pharm Res. (2017) 40:500–8. doi: 10.1007/s12272-017-0902-5 28229391

[98] Yin J Yu C Yang Z He JL Chen WJ Liu HZ. Tetramethylpyrazine inhibits migration of SKOV3 human ovarian carcinoma cells and decreases the expression of interleukin-8 via the ERK1/2, p38 and AP-1 signaling pathways. Oncol Rep. (2011) 26:671–9. doi: 10.3892/or.2011.1334 21637924

[99] Ren MX Deng XH Ai F Yuan GY Song HY. Effect of quercetin on the proliferation of the human ovarian cancer cell line SKOV-3 in vitro. Exp Ther Med. (2015) 10:579–83. doi: 10.3892/etm.2015.2536 PMC4508991 26622357

[100] Zhou J Gong J Ding C Chen G. Quercetin induces the apoptosis of human ovarian carcinoma cells by upregulating the expression of microRNA-145. Mol Med Rep. (2015) 12:3127–31. doi: 10.3892/mmr.2015.3679 25937243

[101] Teekaraman D Elayapillai SP Viswanathan MP Jagadeesan A. Quercetin inhibits human metastatic ovarian cancer cell growth and modulates components of the intrinsic apoptotic pathway in PA-1 cell line. Chem Biol Interact. (2019) 300:91–100. doi: 10.1016/j.cbi.2019.01.008 30639267

[102] Yi L Zongyuan Y Cheng G Lingyun Z Guilian Y Wei G. Quercetin enhances apoptotic effect of tumor necrosis factor-related apoptosis-inducing ligand (TRAIL) in ovarian cancer cells through reactive oxygen species (ROS) mediated CCAAT enhancer-binding protein homologous protein (CHOP)-death receptor 5 pathway. Cancer Sci. (2014) 105:520–7. doi: 10.1111/cas.12395 PMC4317845 24612139

[103] Vergara D Simeone P Toraldo D Del Boccio P Vergaro V Leporatti S. Resveratrol downregulates Akt/GSK and ERK signalling pathways in OVCAR-3 ovarian cancer cells. Mol Biosyst. (2012) 8:1078–87. doi: 10.1039/c2mb05486h 22234583

[104] Mikula-Pietrasik J Sosinska P Ksiazek K. Resveratrol inhibits ovarian cancer cell adhesion to peritoneal mesothelium in *vitro* by modulating the production of alpha5beta1 integrins and hyaluronic acid. Gynecol Oncol. (2014) 134:624–30. doi: 10.1016/j.ygyno.2014.06.022 24995580

[105] Park SY Jeong KJ Lee J Yoon DS Choi WS Kim YK. Hypoxia enhances LPA-induced HIF-1alpha and VEGF expression: their inhibition by resveratrol. Cancer Lett. (2007) 258:63–9. doi: 10.1016/j.canlet.2007.08.011 17919812

[106] Kim SC Choi B Kwon Y. Thiol-reducing agents prevent sulforaphane-induced growth inhibition in ovarian cancer cells. Food Nutr Res. (2017) 61:1368321. doi: 10.1080/16546628.2017.1368321 28970779 PMC5614215

[107] Park S Lim W Jeong W Bazer FW Lee D Song G. Sideroxylin (Callistemon lanceolatus) suppressed cell proliferation and increased apoptosis in ovarian cancer cells accompanied by mitochondrial dysfunction, the generation of reactive oxygen species, and an increase of lipid peroxidation. J Cell Physiol. (2018) 233:8597–604. doi: 10.1002/jcp.26540 29904922

[108] Lee D Ko H Kim YJ Kim SN Choi KC Yamabe N. Inhibition of A2780 human ovarian carcinoma cell proliferation by a rubus component, sanguiin H-6. J Agric Food Chem. (2016) 64:801–5. doi: 10.1021/acs.jafc.5b05461 26725849

[109] Ruibin J Bo J Danying W Chihong Z Jianguo F Linhui G. Therapy effects of wogonin on ovarian cancer cells. BioMed Res Int. (2017) 2017:9381513. doi: 10.1155/2017/9381513 29181409 PMC5664191

[110] Zhao Q Chang W Chen R Liu Y. Anti-proliferative effect of wogonin on ovary cancer cells involves activation of apoptosis and cell cycle arrest. Med Sci Monit. (2019) 25:8465–71. doi: 10.12659/MSM.917823 PMC6865227 31707402

[111] Yang L Zheng XL Sun H Zhong YJ Wang Q He HN. Catalase suppression-mediated H(2)O(2) accumulation in cancer cells by wogonin effectively blocks tumor necrosis factor-induced NF-kappaB activation and sensitizes apoptosis. Cancer Sci. (2011) 102:870–6. doi: 10.1111/j.1349-7006.2011.01874.x 21244577

[112] Chen SS Corteling R Stevanato L Sinden J. Polyphenols inhibit indoleamine 3,5-dioxygenase-1 enzymatic activity–a role of immunomodulation in chemoprevention. Discovery Med. (2012) 14:327–33. 23200064

[113] Kim KK Singh AP Singh RK Demartino A Brard L Vorsa N. Anti-angiogenic activity of cranberry proanthocyanidins and cytotoxic properties in ovarian cancer cells. Int J Oncol. (2012) 40:227–35. doi: 10.3892/ijo.2011.1198 21922132

[114] Liu J Bai J Jiang G Li X Wang J Wu D. Anti-Tumor Effect of Pinus massoniana Bark Proanthocyanidins on Ovarian Cancer through Induction of Cell Apoptosis and Inhibition of Cell Migration. PloS One. (2015) 10:e0142157. doi: 10.1371/journal.pone.0142157 26539720 PMC4634942

[115] Opipari AW Jr. Tan L Boitano AE Sorenson DR Aurora A Liu JR. Resveratrol-induced autophagocytosis in ovarian cancer cells. Cancer Res. (2004) 64:696–703. doi: 10.1158/0008-5472.CAN-03-2404 14744787

[116] Zhong LX Zhang Y Wu ML Liu YN Zhang P Chen XY. Resveratrol and STAT inhibitor enhance autophagy in ovarian cancer cells. Cell Death Discovery. (2016) 2:15071. doi: 10.1038/cddiscovery.2015.71 27551495 PMC4979504

[117] Zhong LX Nie JH Liu J Lin LZ. Correlation of ARHI upregulation with growth suppression and STAT3 inactivation in resveratrol-treated ovarian cancer cells. Cancer biomark. (2018) 21:787–95. doi: 10.3233/CBM-170483 PMC13078328 29504523

[118] Ferraresi A Esposito A Girone C Vallino L Salwa A Ghezzi I. Resveratrol contrasts LPA-induced ovarian cancer cell migration and platinum resistance by rescuing hedgehog-mediated autophagy. Cells. (2021) 10:3213. doi: 10.3390/cells10113213 34831435 PMC8625920

[119] Lang F Qin Z Li F Zhang H Fang Z Hao E. Apoptotic cell death induced by resveratrol is partially mediated by the autophagy pathway in human ovarian cancer cells. PloS One. (2015) 10:e0129196. doi: 10.1371/journal.pone.0129196 26067645 PMC4466135

[120] Fong MY Jin S Rane M Singh RK Gupta R Kakar SS. Withaferin A synergizes the therapeutic effect of doxorubicin through ROS-mediated autophagy in ovarian cancer. PloS One. (2012) 7:e42265. doi: 10.1371/journal.pone.0042265 22860102 PMC3408484

[121] Liu LD Pang YX Zhao XR Li R Jin CJ Xue J. Curcumin induces apoptotic cell death and protective autophagy by inhibiting AKT/mTOR/p70S6K pathway in human ovarian cancer cells. Arch Gynecol Obstet. (2019) 299:1627–39. doi: 10.1007/s00404-019-05058-3 31006841

[122] Zheng X Chen W Hou H Li J Li H Sun X. Ginsenoside 20(S)-Rg3 induced autophagy to inhibit migration and invasion of ovarian cancer. BioMed Pharmacother. (2017) 85:620–6. doi: 10.1016/j.biopha.2016.11.072 27899249

[123] Li J Liu T Zhao L Chen W Hou H Ye Z. Ginsenoside 20(S)−Rg3 inhibits the Warburg effect through STAT3 pathways in ovarian cancer cells. Int J Oncol. (2015) 46:775–81. doi: 10.3892/ijo.2014.2767 25405516

[124] Zheng X Zhou Y Chen W Chen L Lu J He F. Ginsenoside 20(S)-rg3 prevents PKM2-targeting miR-324-5p from H19 sponging to antagonize the warburg effect in ovarian cancer cells. Cell Physiol Biochem. (2018) 51:1340–53. doi: 10.1159/000495552 30481782

[125] Lu J Wang L Chen W Wang Y Zhen S Chen H. miR-603 targeted hexokinase-2 to inhibit the Malignancy of ovarian cancer cells. Arch Biochem Biophys. (2019) 661:1–9. doi: 10.1016/j.abb.2018.10.014 30365936

[126] Gwak H Kim S Dhanasekaran DN Song YS. Resveratrol triggers ER stress-mediated apoptosis by disrupting N-linked glycosylation of proteins in ovarian cancer cells. Cancer Lett. (2016) 371:347–53. doi: 10.1016/j.canlet.2015.11.032 26704305

[127] Tino AB Chitcholtan K Sykes PH Garrill A. Resveratrol and acetyl-resveratrol modulate activity of VEGF and IL-8 in ovarian cancer cell aggregates via attenuation of the NF-kappaB protein. J Ovarian Res. (2016) 9:84. doi: 10.1186/s13048-016-0293-0 27906095 PMC5134119

[128] Chen J Huang ST Chen JG He JH Lin WM Huang ZH. Resveratrol reduces lactate production and modifies the ovarian cancer immune microenvironment. Neoplasma. (2022) 69:1129–37. doi: 10.4149/neo_2022_220414N410 36131607

[129] Li J Zou Y Pei M Zhang Y Jiang Y. Berberine inhibits the Warburg effect through TET3/miR-145/HK2 pathways in ovarian cancer cells. J Cancer. (2021) 12:207–16. doi: 10.7150/jca.48896 PMC7738813 33391417

[130] Yallapu MM Maher DM Sundram V Bell MC Jaggi M Chauhan SC. Curcumin induces chemo/radio-sensitization in ovarian cancer cells and curcumin nanoparticles inhibit ovarian cancer cell growth. J Ovarian Res. (2010) 3:11. doi: 10.1186/1757-2215-3-11 20429876 PMC2880315

[131] Zhang J Liu J Xu X Li L. Curcumin suppresses cisplatin resistance development partly via modulating extracellular vesicle-mediated transfer of MEG3 and miR-214 in ovarian cancer. Cancer Chemother Pharmacol. (2017) 79:479–87. doi: 10.1007/s00280-017-3238-4 28175963

[132] Muhanmode Y Wen MK Maitinuri A Shen G. Curcumin and resveratrol inhibit chemoresistance in cisplatin-resistant epithelial ovarian cancer cells via targeting P13K pathway. Hum Exp Toxicol. (2022) 41:9603271221095929. doi: 10.1177/09603271221095929 35722665

[133] Huang SL Chang TC Sun NK. Curcumin reduces paclitaxel resistance in ovarian carcinoma cells by upregulating SNIP1 and inhibiting NFkappaB activity. Biochem Pharmacol. (2023) 212:115581. doi: 10.1016/j.bcp.2023.115581 37146834

[134] Liu Y Shen Z Zhu T Lu W Fu Y. Curcumin enhances the anti-cancer efficacy of paclitaxel in ovarian cancer by regulating the miR-9-5p/BRCA1 axis. Front Pharmacol. (2022) 13:1014933. doi: 10.3389/fphar.2022.1014933 36703740 PMC9871306

[135] Engelke LH Hamacher A Proksch P Kassack MU. Ellagic acid and resveratrol prevent the development of cisplatin resistance in the epithelial ovarian cancer cell line A2780. J Cancer. (2016) 7:353–63. doi: 10.7150/jca.13754 PMC4749356 26918049

[136] Hasan AA Kalinina E Nuzhina J Volodina Y Shtil A Tatarskiy V. Potentiation of cisplatin cytotoxicity in resistant ovarian cancer SKOV3/cisplatin cells by quercetin pre-treatment. Int J Mol Sci. (2023) 24:10960. doi: 10.3390/ijms241310960 37446140 PMC10341453

[137] Li S Yi Z Li M Zhu Z. Baicalein improves the chemoresistance of ovarian cancer through regulation of CirSLC7A6. J Ovarian Res. (2023) 16:212. doi: 10.1186/s13048-023-01285-0 37940982 PMC10631197

[138] Marverti G Ligabue A Lombardi P Ferrari S Monti MG Frassineti C. Modulation of the expression of folate cycle enzymes and polyamine metabolism by berberine in cisplatin-sensitive and -resistant human ovarian cancer cells. Int J Oncol. (2013) 43:1269–80. doi: 10.3892/ijo.2013.2045 23903781

[139] Liu S Fang Y Shen H Xu W Li H. Berberine sensitizes ovarian cancer cells to cisplatin through miR-21/PDCD4 axis. Acta Biochim Biophys Sin (Shanghai). (2013) 45:756–62. doi: 10.1093/abbs/gmt075 23824073

[140] Zhao Y Cui L Pan Y Shao D Zheng X Zhang F. Berberine inhibits the chemotherapy-induced repopulation by suppressing the arachidonic acid metabolic pathway and phosphorylation of FAK in ovarian cancer. Cell Prolif. (2017) 50:e12393. doi: 10.1111/cpr.12393 28990249 PMC6529084

[141] Zhao Y Yang X Zhao J Gao M Zhang M Shi T. Berberine inhibits chemotherapy-exacerbated ovarian cancer stem cell-like characteristics and metastasis through GLI1. Eur J Pharmacol. (2021) 895:173887. doi: 10.1016/j.ejphar.2021.173887 33482182

[142] Go WJ Ryu JH Qiang F Han HK. Evaluation of the flavonoid oroxylin A as an inhibitor of P-glycoprotein-mediated cellular efflux. J Nat Prod. (2009) 72:1616–9. doi: 10.1021/np9003036 19739602

[143] Chen H Landen CN Li Y Alvarez RD Tollefsbol TO. Enhancement of Cisplatin-Mediated Apoptosis in Ovarian Cancer Cells through Potentiating G2/M Arrest and p21 Upregulation by Combinatorial Epigallocatechin Gallate and Sulforaphane. J Oncol. (2013) 2013:872957. doi: 10.1155/2013/872957 23476648 PMC3588178

[144] Wang X Jiang P Wang P Yang CS Wang X Feng Q. EGCG enhances cisplatin sensitivity by regulating expression of the copper and cisplatin influx transporter CTR1 in ovary cancer. PloS One. (2015) 10:e0125402. doi: 10.1371/journal.pone.0125402 25927922 PMC4416002

[145] Chen H Landen CN Li Y Alvarez RD Tollefsbol TO. Epigallocatechin gallate and sulforaphane combination treatment induce apoptosis in paclitaxel-resistant ovarian cancer cells through hTERT and Bcl-2 down-regulation. Exp Cell Res. (2013) 319:697–706. doi: 10.1016/j.yexcr.2012.12.026 23333498 PMC3577973

[146] Chen S Cooper M Jones M Madhuri TK Wade J Bachelor A. Combined activity of oridonin and wogonin in advanced-stage ovarian cancer cells: sensitivity of ovarian cancer cells to phyto-active chemicals. Cell Biol Toxicol. (2011) 27:133–47. doi: 10.1007/s10565-010-9176-0 20872277

[147] Xing F Sun C Luo N He Y Chen M Ding S. Wogonin increases cisplatin sensitivity in ovarian cancer cells through inhibition of the phosphatidylinositol 3-kinase (PI3K)/Akt pathway. Med Sci Monit. (2019) 25:6007–14. doi: 10.12659/MSM.913829 PMC6703084 31402794

[148] Vinay DS Ryan EP Pawelec G Talib WH Stagg J Elkord E. Immune evasion in cancer: Mechanistic basis and therapeutic strategies. Semin Cancer Biol. (2015) 35 Suppl:S185–S98. doi: 10.1016/j.semcancer.2015.03.004 25818339

[149] Zhang Y Chen S Wei C Rankin GO Ye X Chen YC. Dietary compound proanthocyanidins from Chinese bayberry (Myrica rubra Sieb. et Zucc.) leaves attenuate chemotherapy-resistant ovarian cancer stem cell traits via targeting the Wnt/beta-catenin signaling pathway and inducing G1 cell cycle arrest. Food Funct. (2018) 9:525–33. doi: 10.1039/C7FO01453H PMC5962270 29256569

[150] Zhang Y Chen S Wei C Rankin GO Rojanasakul Y Ren N. Dietary Compound Proanthocyanidins from Chinese bayberry (Myrica rubra Sieb. et Zucc.) leaves inhibit angiogenesis and regulate cell cycle of cisplatin-resistant ovarian cancer cells via targeting Akt pathway. J Funct Foods. (2018) 40:573–81. doi: 10.1016/j.jff.2017.11.045 PMC5863932 29576805

[151] Pellegrini E Multari G Gallo FR Vecchiotti D Zazzeroni F Condello M. A natural product, voacamine, sensitizes paclitaxel-resistant human ovarian cancer cells. Toxicol Appl Pharmacol. (2022) 434:115816. doi: 10.1016/j.taap.2021.115816 34856211

[152] Wang G Li L Li Y Zhang LH. Toosendanin reduces cisplatin resistance in ovarian cancer through modulating the miR-195/ERK/beta-catenin pathway. Phytomedicine. (2023) 109:154571. doi: 10.1016/j.phymed.2022.154571 36610147

[153] Zhang T Xu C Zheng P Zhang X Qiu C Wu F. Glaucocalyxin B attenuates ovarian cancer cell growth and cisplatin resistance *in vitro* via activating oxidative stress. Oxid Med Cell Longev. (2022) 2022:6324292. doi: 10.1155/2022/6324292 35251480 PMC8896941

[154] Yun UJ Lee JH Koo KH Ye SK Kim SY Lee CH. Lipid raft modulation by Rp1 reverses multidrug resistance via inactivating MDR-1 and Src inhibition. Biochem Pharmacol. (2013) 85:1441–53. doi: 10.1016/j.bcp.2013.02.025 23473805

[155] Deng S Wong CKC Lai HC Wong AST. Ginsenoside-Rb1 targets chemotherapy-resistant ovarian cancer stem cells via simultaneous inhibition of Wnt/beta-catenin signaling and epithelial-to-mesenchymal transition. Oncotarget. (2017) 8:25897–914. doi: 10.18632/oncotarget.v8i16 PMC5432225 27825116

[156] Barani M Bilal M Sabir F Rahdar A Kyzas GZ. Nanotechnology in ovarian cancer: Diagnosis and treatment. Life Sci. (2021) 266:118914. doi: 10.1016/j.lfs.2020.118914 33340527

[157] Vandghanooni S Eskandani M Barar J Omidi Y. AS1411 aptamer-decorated cisplatin-loaded poly(lactic-co-glycolic acid) nanoparticles for targeted therapy of miR-21-inhibited ovarian cancer cells. Nanomedicine (Lond). (2018) 13:2729–58. doi: 10.2217/nnm-2018-0205 30394201

[158] Fang X Xu Y Wang S Wan J He C Chen M. Pluronic F68-linoleic acid nano-spheres mediated delivery of gambogic acid for cancer therapy. AAPS PharmSciTech. (2017) 18:147–55. doi: 10.1208/s12249-015-0473-z 26912357

[159] Tang F Li L Chen D. Mesoporous silica nanoparticles: synthesis, biocompatibility and drug delivery. Adv Mater. (2012) 24:1504–34. doi: 10.1002/adma.201104763 22378538

[160] Chrastina A Massey KA Schnitzer JE. Overcoming in *vivo* barriers to targeted nanodelivery. Wiley Interdiscip Rev Nanomed Nanobiotechnol. (2011) 3:421–37. doi: 10.1002/wnan.143 21538941

[161] Yao S Li L Su XT Wang K Lu ZJ Yuan CZ. Development and evaluation of novel tumor-targeting paclitaxel-loaded nano-carriers for ovarian cancer treatment: in *vitro* and in vivo. J Exp Clin Cancer Res. (2018) 37:29. doi: 10.1186/s13046-018-0700-z 29478415 PMC6389131

[162] Li Y Gao Y Zhang X Guo H Gao H. Nanoparticles in precision medicine for ovarian cancer: From chemotherapy to immunotherapy. Int J Pharm. (2020) 591:119986. doi: 10.1016/j.ijpharm.2020.119986 33069895

[163] Frank D Indermun S Govender M Kumar P Choonara YE du Toit LC. Antineoplastic nano-lipobubbles for passively targeted ovarian cancer therapy. Colloids Surf B Biointerfaces. (2019) 177:160–8. doi: 10.1016/j.colsurfb.2019.01.049 30731392

[164] Fan Y Marioli M Zhang K. Analytical characterization of liposomes and other lipid nanoparticles for drug delivery. J Pharm BioMed Anal. (2021) 192:113642. doi: 10.1016/j.jpba.2020.113642 33011580

[165] Tenchov R Bird R Curtze AE Zhou Q. Lipid Nanoparticles horizontal line From Liposomes to mRNA Vaccine Delivery, a Landscape of Research Diversity and Advancement. ACS Nano. (2021) 15:16982–7015. doi: 10.1021/acsnano.1c04996 34181394

[166] Tenchov R Sasso JM Wang X Liaw WS Chen CA Zhou QA. Exosomes horizontal line Nature’s Lipid Nanoparticles, a Rising Star in Drug Delivery and Diagnostics. ACS Nano. (2022) 16:17802–46. doi: 10.1021/acsnano.2c08774 PMC9706680 36354238

[167] Srivastava A Babu A Filant J Moxley KM Ruskin R Dhanasekaran D. Exploitation of exosomes as nanocarriers for gene-, chemo-, and immune-therapy of cancer. J BioMed Nanotechnol. (2016) 12:1159–73. doi: 10.1166/jbn.2016.2205 27319211

[168] Croft PK Sharma S Godbole N Rice GE Salomon C. Ovarian-cancer-associated extracellular vesicles: microenvironmental regulation and potential clinical applications. Cells. (2021) 10:2272. doi: 10.3390/cells10092272 34571921 PMC8471580

[169] Wiklander OP Nordin JZ O’Loughlin A Gustafsson Y Corso G Mager I. Extracellular vesicle in *vivo* biodistribution is determined by cell source, route of administration and targeting. J Extracell Vesicles. (2015) 4:26316. doi: 10.3402/jev.v4.26316 25899407 PMC4405624

[170] Wen C Seeger RC Fabbri M Wang L Wayne AS Jong AY. Biological roles and potential applications of immune cell-derived extracellular vesicles. J Extracell Vesicles. (2017) 6:1400370. doi: 10.1080/20013078.2017.1400370 29209467 PMC5706476

[171] Munagala R Aqil F Jeyabalan J Agrawal AK Mudd AM Kyakulaga AH. Exosomal formulation of anthocyanidins against multiple cancer types. Cancer Lett. (2017) 393:94–102. doi: 10.1016/j.canlet.2017.02.004 28202351 PMC5837866

[172] Slavin JL Lloyd B. Health benefits of fruits and vegetables. Adv Nutr. (2012) 3:506–16. doi: 10.3945/an.112.002154 PMC3649719 22797986

[173] Stoner GD. Foodstuffs for preventing cancer: the preclinical and clinical development of berries. Cancer Prev Res (Phila). (2009) 2:187–94. doi: 10.1158/1940-6207.CAPR-08-0226 PMC2769015 19258544

[174] Aqil F Jeyabalan J Agrawal AK Kyakulaga AH Munagala R Parker L. Exosomal delivery of berry anthocyanidins for the management of ovarian cancer. Food Funct. (2017) 8:4100–7. doi: 10.1039/C7FO00882A 28991298

[175] Liu H Shen M Zhao D Ru D Duan Y Ding C. The effect of triptolide-loaded exosomes on the proliferation and apoptosis of human ovarian cancer SKOV3 cells. BioMed Res Int. (2019) 2019:2595801. doi: 10.1155/2019/2595801 31240207 PMC6556367

[176] Melzer C Rehn V Yang Y Bahre H von der Ohe J Hass R. Taxol-loaded MSC-derived exosomes provide a therapeutic vehicle to target metastatic breast cancer and other carcinoma cells. Cancers (Basel). (2019) 11:798. doi: 10.3390/cancers11060798 31181850 PMC6627807

[177] van Niel G D’Angelo G Raposo G. Shedding light on the cell biology of extracellular vesicles. Nat Rev Mol Cell Biol. (2018) 19:213–28. doi: 10.1038/nrm.2017.125 29339798

[178] Minciacchi VR Freeman MR Di Vizio D. Extracellular vesicles in cancer: exosomes, microvesicles and the emerging role of large oncosomes. Semin Cell Dev Biol. (2015) 40:41–51. doi: 10.1016/j.semcdb.2015.02.010 25721812 PMC4747631

[179] Ghasemzadeh T Hasannia M Abnous K Taghdisi SM Nekooei S Nekooei N. Preparation of targeted theranostic red blood cell membranes-based nanobubbles for treatment of colon adenocarcinoma. Expert Opin Drug Deliv. (2023) 20:131–43. doi: 10.1080/17425247.2022.2152792 36427011

[180] Pascucci L Cocce V Bonomi A Ami D Ceccarelli P Ciusani E. Paclitaxel is incorporated by mesenchymal stromal cells and released in exosomes that inhibit in *vitro* tumor growth: a new approach for drug delivery. J Control Release. (2014) 192:262–70. doi: 10.1016/j.jconrel.2014.07.042 25084218

[181] Stompor-Goracy M Bajek-Bil A Machaczka M. Chrysin: perspectives on contemporary status and future possibilities as pro-health agent. Nutrients. (2021) 13:2038. doi: 10.3390/nu13062038 34198618 PMC8232110

[182] Naz S Imran M Rauf A Orhan IE Shariati MA Iahtisham Ul H. Chrysin: Pharmacological and therapeutic properties. Life Sci. (2019) 235:116797. doi: 10.1016/j.lfs.2019.116797 31472146

[183] Tarahomi M Firouzi Amandi A Eslami M Yazdani Y Salek Farrokhi A Ghorbani F. Niosomes nanoparticles as a novel approach in drug delivery enhances anticancer properties of chrysin in human ovarian carcinoma cells (SKOV3): an in *vitro* study. Med Oncol. (2023) 40:87. doi: 10.1007/s12032-023-01952-8 36723692

[184] Bondi ML Emma MR Botto C Augello G Azzolina A Di Gaudio F. Biocompatible lipid nanoparticles as carriers to improve curcumin efficacy in ovarian cancer treatment. J Agric Food Chem. (2017) 65:1342–52. doi: 10.1021/acs.jafc.6b04409 28111949

[185] Zhang X Lu T Ma Y Li R Pang Y Mao H. Novel nanocomplexes targeting STAT3 demonstrate promising anti-ovarian cancer effects *in vivo* . Onco Targets Ther. (2020) 13:5069–82. doi: 10.2147/OTT.S247398 PMC7292488 32606729

[186] Sivadasan D Ramakrishnan K Mahendran J Ranganathan H Karuppaiah A Rahman H. Solid lipid nanoparticles: applications and prospects in cancer treatment. Int J Mol Sci. (2023) 24:6199. doi: 10.3390/ijms24076199 37047172 PMC10094605

[187] Tarr BD Sambandan TG Yalkowsky SH. A new parenteral emulsion for the administration of taxol. Pharm Res. (1987) 4:162–5. doi: 10.1023/A:1016483406511 2908138

[188] Lee MK Lim SJ Kim CK. Preparation, characterization and in *vitro* cytotoxicity of paclitaxel-loaded sterically stabilized solid lipid nanoparticles. Biomaterials. (2007) 28:2137–46. doi: 10.1016/j.biomaterials.2007.01.014 17257668

[189] Senthil Kumar C Thangam R Mary SA Kannan PR Arun G Madhan B. Targeted delivery and apoptosis induction of trans-resveratrol-ferulic acid loaded chitosan coated folic acid conjugate solid lipid nanoparticles in colon cancer cells. Carbohydr Polym. (2020) 231:115682. doi: 10.1016/j.carbpol.2019.115682 31888816

[190] Shariatinia Z. Carboxymethyl chitosan: Properties and biomedical applications. Int J Biol Macromol. (2018) 120:1406–19. doi: 10.1016/j.ijbiomac.2018.09.131 30267813

[191] Chamani M Maleki Dana P Chaichian S Moazzami B Asemi Z. Chitosan is a potential inhibitor of ovarian cancer: Molecular aspects. IUBMB Life. (2020) 72:687–97. doi: 10.1002/iub.2206 31873986

[192] Han HD Mangala LS Lee JW Shahzad MM Kim HS Shen D. Targeted gene silencing using RGD-labeled chitosan nanoparticles. Clin Cancer Res. (2010) 16:3910–22. doi: 10.1158/1078-0432.CCR-10-0005 PMC2912984 20538762

[193] Peter S Alven S Maseko RB Aderibigbe BA. Doxorubicin-based hybrid compounds as potential anticancer agents: A review. Molecules. (2022) 27:4478. doi: 10.3390/molecules27144478 35889350 PMC9318127

[194] Hu R Zheng H Cao J Davoudi Z Wang Q. Synthesis and *in vitro* characterization of carboxymethyl chitosan-CBA-doxorubicin conjugate nanoparticles as pH-sensitive drug delivery systems. J BioMed Nanotechnol. (2017) 13:1097–105. doi: 10.1166/jbn.2017.2407 31251142

[195] Yan E Fan Y Sun Z Gao J Hao X Pei S. Biocompatible core-shell electrospun nanofibers as potential application for chemotherapy against ovary cancer. Mater Sci Eng C Mater Biol Appl. (2014) 41:217–23. doi: 10.1016/j.msec.2014.04.053 24907754

[196] Javid A Ahmadian S Saboury AA Kalantar SM Rezaei-Zarchi S. Chitosan-coated superparamagnetic iron oxide nanoparticles for doxorubicin delivery: synthesis and anticancer effect against human ovarian cancer cells. Chem Biol Drug Des. (2013) 82:296–306. doi: 10.1111/cbdd.12145 23594157

[197] Yousefpour P Atyabi F Vasheghani-Farahani E Movahedi AA Dinarvand R. Targeted delivery of doxorubicin-utilizing chitosan nanoparticles surface-functionalized with anti-Her2 trastuzumab. Int J Nanomedicine. (2011) 6:1977–90. doi: 10.2147/IJN.S21523 PMC3181058 21976974

[198] Li X Kong X Zhang J Wang Y Wang Y Shi S. A novel composite hydrogel based on chitosan and inorganic phosphate for local drug delivery of camptothecin nanocolloids. J Pharm Sci. (2011) 100:232–41. doi: 10.1002/jps.22256 20533555

[199] Zhou L Du L Chen X Li X Li Z Wen Y. The antitumor and antimetastatic effects of N-trimethyl chitosan-encapsulated camptothecin on ovarian cancer with minimal side effects. Oncol Rep. (2010) 24:941–8. doi: 10.3892/or.2010.941 20811674

[200] Cortes JR Perez GM Rivas MD Zamorano J. Kaempferol inhibits IL-4-induced STAT6 activation by specifically targeting JAK3. J Immunol. (2007) 179:3881–7. doi: 10.4049/jimmunol.179.6.3881 17785825

[201] Duthie G Crozier A. Plant-derived phenolic antioxidants. Curr Opin Clin Nutr Metab Care. (2000) 3:447–51. doi: 10.1097/00075197-200011000-00006 11085830

[202] Luo H Jiang B Li B Li Z Jiang BH Chen YC. Kaempferol nanoparticles achieve strong and selective inhibition of ovarian cancer cell viability. Int J Nanomedicine. (2012) 7:3951–9. doi: 10.2147/IJN.S33670 PMC3410694 22866004

[203] Dong X Zeng Y Zhang Z Fu J You L He Y. Hypericin-mediated photodynamic therapy for the treatment of cancer: a review. J Pharm Pharmacol. (2021) 73:425–36. doi: 10.1093/jpp/rgaa018 33793828

[204] Zeisser-Labouebe M Lange N Gurny R Delie F. Hypericin-loaded nanoparticles for the photodynamic treatment of ovarian cancer. Int J Pharm. (2006) 326:174–81. doi: 10.1016/j.ijpharm.2006.07.012 16930882

[205] Mukund V Mukund D Sharma V Mannarapu M Alam A. Genistein: Its role in metabolic diseases and cancer. Crit Rev Oncol Hematol. (2017) 119:13–22. doi: 10.1016/j.critrevonc.2017.09.004 29065980

[206] Patra A Satpathy S Naik PK Kazi M Hussain MD. Folate receptor-targeted PLGA-PEG nanoparticles for enhancing the activity of genistein in ovarian cancer. Artif Cells Nanomed Biotechnol. (2022) 50:228–39. doi: 10.1080/21691401.2022.2118758 36330543

[207] Kumar SS Surianarayanan M Vijayaraghavan R Mandal AB MacFarlane DR. Curcumin loaded poly(2-hydroxyethyl methacrylate) nanoparticles from gelled ionic liquid–*in vitro* cytotoxicity and anti-cancer activity in SKOV-3 cells. Eur J Pharm Sci. (2014) 51:34–44. doi: 10.1016/j.ejps.2013.08.036 24012589

[208] Yallapu MM Gupta BK Jaggi M Chauhan SC. Fabrication of curcumin encapsulated PLGA nanoparticles for improved therapeutic effects in metastatic cancer cells. J Colloid Interface Sci. (2010) 351:19–29. doi: 10.1016/j.jcis.2010.05.022 20627257

[209] Sandhiutami NMD Arozal W Louisa M Rahmat D Wuyung PE. Curcumin nanoparticle enhances the anticancer effect of cisplatin by inhibiting PI3K/AKT and JAK/STAT3 pathway in rat ovarian carcinoma induced by DMBA. Front Pharmacol. (2020) 11:603235. doi: 10.3389/fphar.2020.603235 33536913 PMC7848208

[210] Mahmud A Xiong XB Aliabadi HM Lavasanifar A. Polymeric micelles for drug targeting. J Drug Targeting. (2007) 15:553–84. doi: 10.1080/10611860701538586 17968711

[211] Talelli M Rijcken CJ van Nostrum CF Storm G Hennink WE. Micelles based on HPMA copolymers. Adv Drug Delivery Rev. (2010) 62:231–9. doi: 10.1016/j.addr.2009.11.029 20004693

[212] Maher P. How fisetin reduces the impact of age and disease on CNS function. Front Biosci (Schol Ed). (2015) 7:58–82. doi: 10.2741/s425 25961687 PMC5527824

[213] Rais J Khan H Arshad M. The role of phytochemicals in cancer prevention: A review with emphasis on baicalein, fisetin, and biochanin A. Curr Top Med Chem. (2023) 23:1123–35. doi: 10.2174/1568026623666230516161827 37194231

[214] Xiao X Zou J Fang Y Meng Y Xiao C Fu J. Fisetin and polymeric micelles encapsulating fisetin exhibit potent cytotoxic effects towards ovarian cancer cells. BMC Complement Altern Med. (2018) 18:91. doi: 10.1186/s12906-018-2127-7 29544480 PMC5855937

[215] Carlson LJ Cote B Alani AW Rao DA. Polymeric micellar co-delivery of resveratrol and curcumin to mitigate in *vitro* doxorubicin-induced cardiotoxicity. J Pharm Sci. (2014) 103:2315–22. doi: 10.1002/jps.24042 24914015

[216] Khiste SK Liu Z Roy KR Uddin MB Hosain SB Gu X. Ceramide-rubusoside nanomicelles, a potential therapeutic approach to target cancers carrying p53 missense mutations. Mol Cancer Ther. (2020) 19:564–74. doi: 10.1158/1535-7163.MCT-19-0366 PMC7007850 31645443

[217] Jiao M Zhang P Meng J Li Y Liu C Luo X. Recent advancements in biocompatible inorganic nanoparticles towards biomedical applications. Biomater Sci. (2018) 6:726–45. doi: 10.1039/C7BM01020F 29308496

[218] Saratale RG Saratale GD Ghodake G Cho SK Kadam A Kumar G. Wheat straw extracted lignin in silver nanoparticles synthesis: Expanding its prophecy towards antineoplastic potency and hydrogen peroxide sensing ability. Int J Biol Macromol. (2019) 128:391–400. doi: 10.1016/j.ijbiomac.2019.01.120 30684583

[219] Rammohan A Gunasekar D Reddy NV Vijaya T Devillee A Bodo B. Structure elucidation and antioxidant activity of the phenolic compounds from Rhynchosia suaveolens. Nat Prod Commun. (2015) 10:609–11. doi: 10.1177/1934578X1501000418 25973488

[220] Bethu MS Netala VR Domdi L Tartte V Janapala VR. Potential anticancer activity of biogenic silver nanoparticles using leaf extract of Rhynchosia suaveolens: an insight into the mechanism. Artif Cells Nanomed Biotechnol. (2018) 46:104–14. doi: 10.1080/21691401.2017.1414824 29301413

[221] Schluep T Gunawan P Ma L Jensen GS Duringer J Hinton S. Polymeric tubulysin-peptide nanoparticles with potent antitumor activity. Clin Cancer Res. (2009) 15:181–9. doi: 10.1158/1078-0432.CCR-08-1848 19118045

[222] Zhang X Haney KM Richardson AC Wilson E Gewirtz DA Ware JL. Anibamine, a natural product CCR5 antagonist, as a novel lead for the development of anti-prostate cancer agents. Bioorg Med Chem Lett. (2010) 20:4627–30. doi: 10.1016/j.bmcl.2010.06.003 PMC2914538 20579875

[223] Jacome Sanz D Raivola J Karvonen H Arjama M Barker H Murumagi A. Evaluating targeted therapies in ovarian cancer metabolism: novel role for PCSK9 and second generation mTOR inhibitors. Cancers (Basel). (2021) 13:3727. doi: 10.3390/cancers13153727 34359627 PMC8345177

[224] Coppinger C Pomales B Movahed MR Marefat M Hashemzadeh M. Berberine: A multi-target natural PCSK9 inhibitor with the potential to treat diabetes, Alzheimer’s, cancer and cardiovascular disease. Curr Rev Clin Exp Pharmacol. (2024) 19:312–26. doi: 10.2174/0127724328250471231222094648 38361373

[225] Balasubramanyam K Varier RA Altaf M Swaminathan V Siddappa NB Ranga U. Curcumin, a novel p300/CREB-binding protein-specific inhibitor of acetyltransferase, represses the acetylation of histone/nonhistone proteins and histone acetyltransferase-dependent chromatin transcription. J Biol Chem. (2004) 279:51163–71. doi: 10.1074/jbc.M409024200 15383533

[226] Soflaei SS Momtazi-Borojeni AA Majeed M Derosa G Maffioli P Sahebkar A. Curcumin: A natural pan-HDAC inhibitor in cancer. Curr Pharm Des. (2018) 24:123–9. doi: 10.2174/1381612823666171114165051 29141538

[227] Rodrigues Moita AJ Bandolik JJ Hansen FK Kurz T Hamacher A Kassack MU. Priming with HDAC inhibitors sensitizes ovarian cancer cells to treatment with cisplatin and HSP90 inhibitors. Int J Mol Sci. (2020) 21:8300. doi: 10.3390/ijms21218300 33167494 PMC7663919

[228] Yang D Huang FX Wei W Li QQ Wu JW Huang Y. Loss of HRD functional phenotype impedes immunotherapy and can be reversed by HDAC inhibitor in ovarian cancer. Int J Biol Sci. (2023) 19:1846–60. doi: 10.7150/ijbs.79654 PMC10092773 37063431

[229] Tossetta G Marzioni D. Natural and synthetic compounds in Ovarian Cancer: A focus on NRF2/KEAP1 pathway. Pharmacol Res. (2022) 183:106365. doi: 10.1016/j.phrs.2022.106365 35901941

[230] Tossetta G Fantone S Montanari E Marzioni D Goteri G. Role of NRF2 in ovarian cancer. Antioxidants (Basel). (2022) 11:663. doi: 10.3390/antiox11040663 35453348 PMC9027335

[231] Li X Zhang Q Hou N Li J Liu M Peng S. Carnosol as a nrf2 activator improves endothelial barrier function through antioxidative mechanisms. Int J Mol Sci. (2019) 20:880. doi: 10.3390/ijms20040880 30781644 PMC6413211

[232] Kim EN Lim JH Kim MY Ban TH Jang IA Yoon HE. Resveratrol, an Nrf2 activator, ameliorates aging-related progressive renal injury. Aging (Albany NY). (2018) 10:83–99. doi: 10.18632/aging.v10i1 29326403 PMC5811244

[233] Moon SJ Jhun J Ryu J Kwon JY Kim SY Jung K. The anti-arthritis effect of sulforaphane, an activator of Nrf2, is associated with inhibition of both B cell differentiation and the production of inflammatory cytokines. PloS One. (2021) 16:e0245986. doi: 10.1371/journal.pone.0245986 33592002 PMC7886167

[234] Chen Y Yang J Zuo Y Zhang C Pu Y Ren Q. Voacamine is a novel inhibitor of EGFR exerting oncogenic activity against colorectal cancer through the mitochondrial pathway. Pharmacol Res. (2022) 184:106415. doi: 10.1016/j.phrs.2022.106415 36029932

[235] Falkenberg KD Jakobs A Matern JC Dorner W Uttarkar S Trentmann A. a natural compound with anti-tumor activity, is a potent inhibitor of transcription factor C/EBPbeta. Biochim Biophys Acta Mol Cell Res. (2017) 1864:1349–58. doi: 10.1016/j.bbamcr.2017.05.003 28476645

[236] Zalpoor H Nabi-Afjadi M Forghaniesfidvajani R Tavakol C Farahighasreaboonasr F Pakizeh F. Quercetin as a JAK-STAT inhibitor: a potential role in solid tumors and neurodegenerative diseases. Cell Mol Biol Lett. (2022) 27:60. doi: 10.1186/s11658-022-00355-3 35883021 PMC9327369

[237] Yao JY Xu S Sun YN Xu Y Guo QL Wei LB. Novel CDK9 inhibitor oroxylin A promotes wild-type P53 stability and prevents hepatocellular carcinoma progression by disrupting both MDM2 and SIRT1 signaling. Acta Pharmacol Sin. (2022) 43:1033–45. doi: 10.1038/s41401-021-00708-2 PMC8975870 34188177

[238] Chen Y Zheng J Mo L Chen F Li R Wang Y. Oroxylin A suppresses breast cancer-induced osteoclastogenesis and osteolysis as a natural RON inhibitor. Phytomedicine. (2024) 129:155688. doi: 10.1016/j.phymed.2024.155688 38728920

[239] Jia L Song Q Zhou C Li X Pi L Ma X. Dihydroartemisinin as a putative STAT3 inhibitor, suppresses the growth of head and neck squamous cell carcinoma by targeting Jak2/STAT3 signaling. PloS One. (2016) 11:e0147157. doi: 10.1371/journal.pone.0147157 26784960 PMC4718674

[240] Ni L Zhu X Zhao Q Shen Y Tao L Zhang J. Dihydroartemisinin, a potential PTGS1 inhibitor, potentiated cisplatin-induced cell death in non-small cell lung cancer through activating ROS-mediated multiple signaling pathways. Neoplasia. (2024) 51:100991. doi: 10.1016/j.neo.2024.100991 38507887 PMC10965827

[241] Huang Q Su H Qi B Wang Y Yan K Wang X. A SIRT1 activator, ginsenoside rc, promotes energy metabolism in cardiomyocytes and neurons. J Am Chem Soc. (2021) 143:1416–27. doi: 10.1021/jacs.0c10836 33439015

[242] Yang Z Yu Y Sun N Zhou L Zhang D Chen H. Ginsenosides Rc, as a novel SIRT6 activator, protects mice against high fat diet induced NAFLD. J Ginseng Res. (2023) 47:376–84. doi: 10.1016/j.jgr.2020.07.005 PMC10214131 37252281

[243] Zhong Y Chen Y Pan Z Tang K Zhong G Guo J. Ginsenoside Rc, as an FXR activator, alleviates acetaminophen-induced hepatotoxicity via relieving inflammation and oxidative stress. Front Pharmacol. (2022) 13:1027731. doi: 10.3389/fphar.2022.1027731 36278209 PMC9585238

[244] Li D Bi FF Chen NN Cao JM Sun WP Zhou YM. A novel crosstalk between BRCA1 and sirtuin 1 in ovarian cancer. Sci Rep. (2014) 4:6666. doi: 10.1038/srep06666 25323003 PMC4200400

[245] Sun X Wang S Li Q. Comprehensive analysis of expression and prognostic value of sirtuins in ovarian cancer. Front Genet. (2019) 10:879. doi: 10.3389/fgene.2019.00879 31572453 PMC6754078

[246] Rezen T Rozman D Kovacs T Kovacs P Sipos A Bai P. The role of bile acids in carcinogenesis. Cell Mol Life Sci. (2022) 79:243. doi: 10.1007/s00018-022-04278-2 35429253 PMC9013344

[247] Cheng Z Li Y Zhao D Zhao W Wu M Zhang W. Nanocarriers for intracellular co-delivery of proteins and small-molecule drugs for cancer therapy. Front Bioeng Biotechnol. (2022) 10:994655. doi: 10.3389/fbioe.2022.994655 36147526 PMC9485877

[248] Guo X Zhao Z Chen D Qiao M Wan F Cun D. Co-delivery of resveratrol and docetaxel via polymeric micelles to improve the treatment of drug-resistant tumors. Asian J Pharm Sci. (2019) 14:78–85. doi: 10.1016/j.ajps.2018.03.002 32104440 PMC7032195

[249] Bose S Banerjee S Mondal A Chakraborty U Pumarol J Croley CR. Targeting the JAK/STAT signaling pathway using phytocompounds for cancer prevention and therapy. Cells. (2020) 9:1451. doi: 10.3390/cells9061451 32545187 PMC7348822

[250] Li Y Wang X Ma X Liu C Wu J Sun C. Natural polysaccharides and their derivates: A promising natural adjuvant for tumor immunotherapy. Front Pharmacol. (2021) 12:621813. doi: 10.3389/fphar.2021.621813 33935714 PMC8080043

[251] Yang M Li J Gu P Fan X. The application of nanoparticles in cancer immunotherapy: Targeting tumor microenvironment. Bioact Mater. (2021) 6:1973–87. doi: 10.1016/j.bioactmat.2020.12.010 PMC7773537 33426371

[252] Wu S Yang X Lu Y Fan Z Li Y Jiang Y. A green approach to dual-drug nanoformulations with targeting and synergistic effects for cancer therapy. Drug Deliv. (2017) 24:51–60. doi: 10.1080/10717544.2016.1228716 28155539 PMC8241172

[253] Fu S Li G Zang W Zhou X Shi K Zhai Y. Pure drug nano-assemblies: A facile carrier-free nanoplatform for efficient cancer therapy. Acta Pharm Sin B. (2022) 12:92–106. doi: 10.1016/j.apsb.2021.08.012 35127374 PMC8799886

