# Supplementary material for: A nanoscale natural drug delivery system for targeted drug delivery against ovarian cancer: action mechanism, application enlightenment and future potential
Source: Front Immunol. 2024 Oct 11;15:1427573. doi: 10.3389/fimmu.2024.1427573 (PMC11502327; doi:10.3389/fimmu.2024.1427573)
Supplement: Supplementary file 1 [file Table_1.docx]

**Supplementary table. The structure of natural drug ingredients.**

**(from https://pubchem.ncbi.nlm.nih.gov/)**

| Class | Phytochemicals | Structure |
| --- | --- | --- |
| Alkaloid | Anibamine | 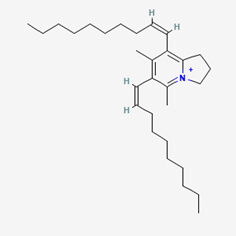 |
|  | Berbamine | 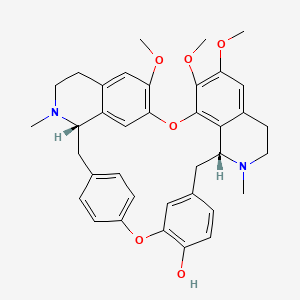 |
|  | Berberine | 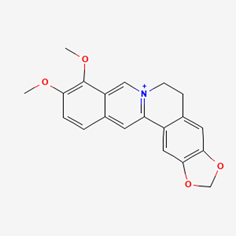 |
|  | Harmine | 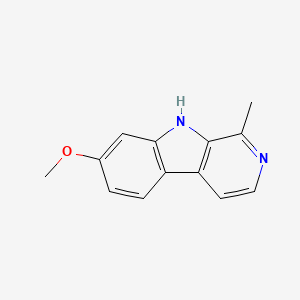 |
|  | Voacamine | 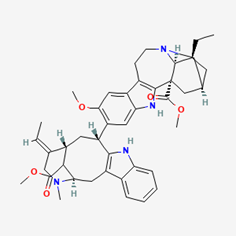 |
| Flavonoid | Amentoflavone | 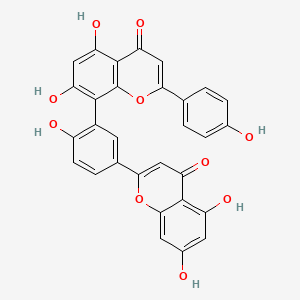 |
|  | Baicalein | 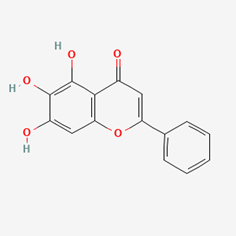 |
|  | Baicalin | 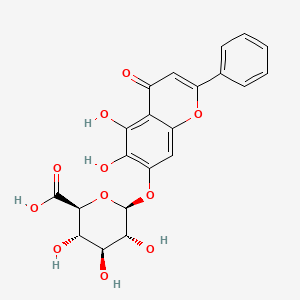 |
|  | Epigallocatechin Gallate | 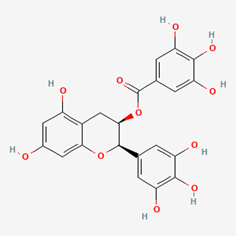 |
|  | Formononetin | 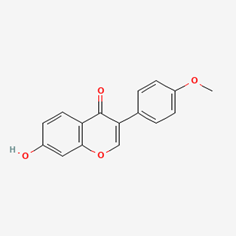 |
|  | Icaritin | 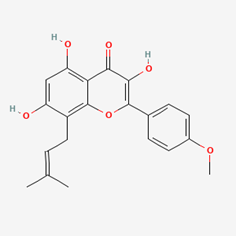 |
|  | Isoliquiritigenin | 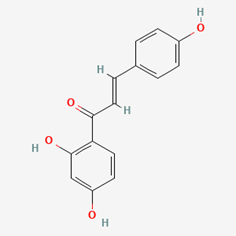 |
|  | Oroxylin A | 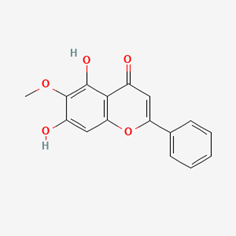 |
|  | Quercetin | 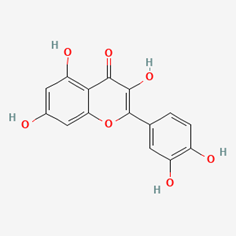 |
|  | Scutellarein | 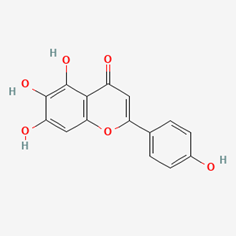 |
|  | Wogonin | 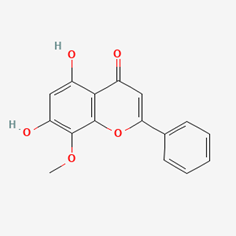 |
| Polyphenol | Curcumin | 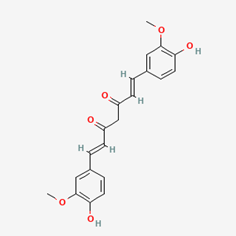 |
|  | Resveratrol | 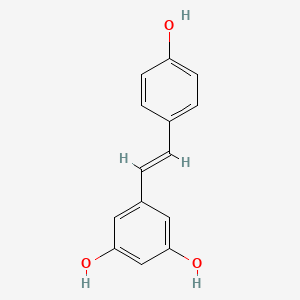 |
|  | Sideroxylin | 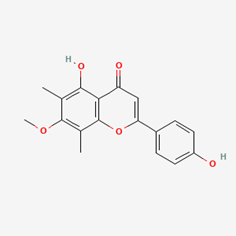 |
|  | Sanguiin H-6 | 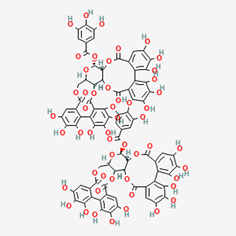 |
|  | proanthocyanidins | 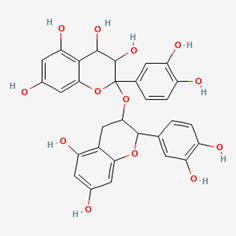 |
| Terpenoid | Asiatic acid | 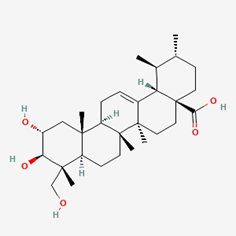 |
|  | Cucurbitacin-A | 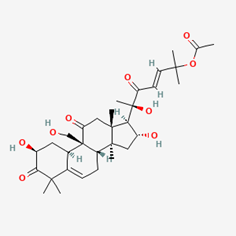 |
|  | Carnosol | 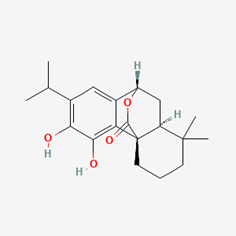 |
|  | Dihydroartemisinin | 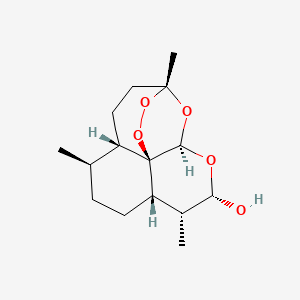 |
|  | Ginsenosides | 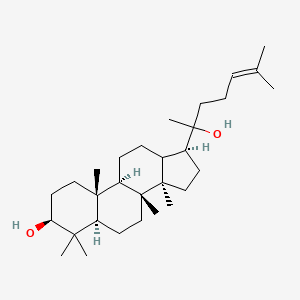 |
|  | Grifolin | 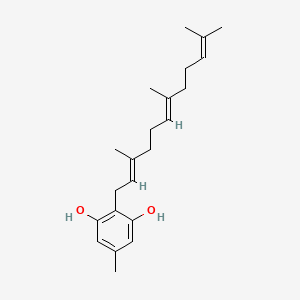 |
|  | Kudsuphilactone B | 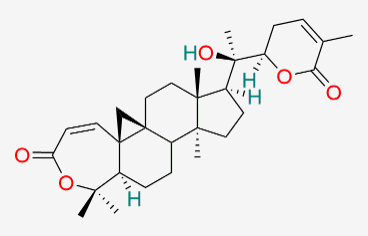 |
|  | Lycopene | 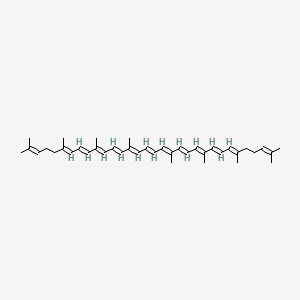 |
|  | Zeylenone | 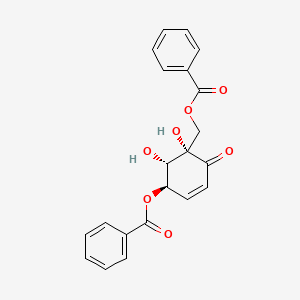 |
|  | Toosendanin | 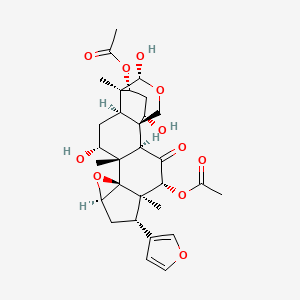 |
|  | Glaucocalyxin B | 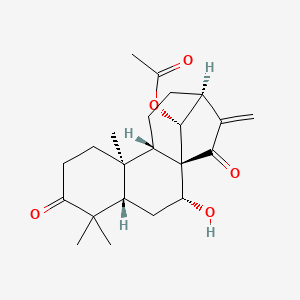 |
| Lactone | Withaferin-A | 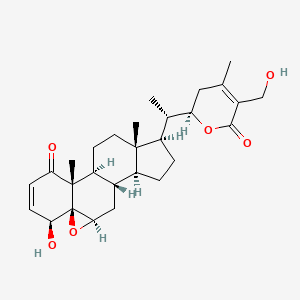 |
| Isothiocyanate | Sulforaphane | 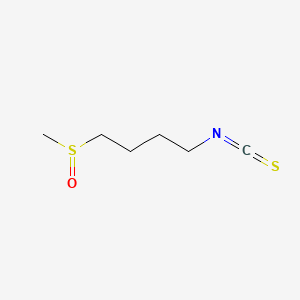 |
| Lignan | Deoxyschizandrin | 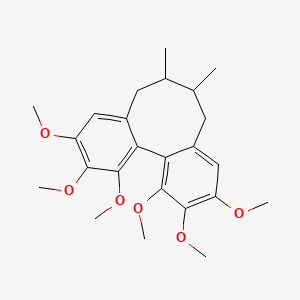 |
| Polysaccharide | Astragalus polysaccharide | 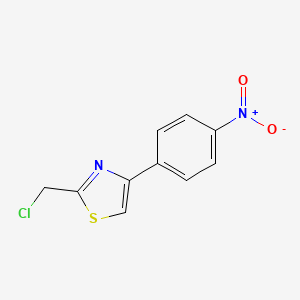 |
| Pyrazine | Tetramethylpyrazine | 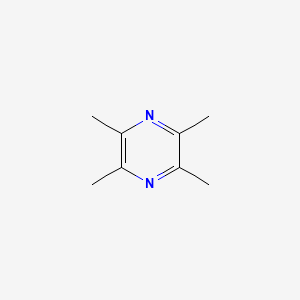 |
| Quinones | Emodin | 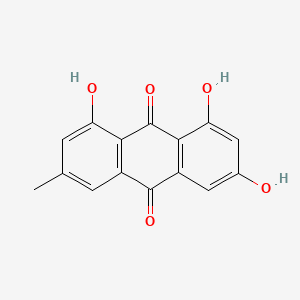 |
